# Assessment of optimal strategies in a two-patch dengue transmission model with seasonality

**DOI:** 10.1371/journal.pone.0173673

**Published:** 2017-03-16

**Authors:** Jung Eun Kim, Hyojung Lee, Chang Hyeong Lee, Sunmi Lee

**Affiliations:** 1 Department of Mathematical Sciences, Ulsan National Institute of Science and Technology, Ulsan, Republic of Korea; 2 Department of Applied Mathematics, Kyung Hee University, Yongin, Republic of Korea; 3 Institute of Natural Sciences, Kyung Hee University, Yongin, Republic of Korea; Columbia University, UNITED STATES

## Abstract

Emerging and re-emerging dengue fever has posed serious problems to public health officials in many tropical and subtropical countries. Continuous traveling in seasonally varying areas makes it more difficult to control the spread of dengue fever. In this work, we consider a two-patch dengue model that can capture the movement of host individuals between and within patches using a residence-time matrix. A previous two-patch dengue model without seasonality is extended by adding host demographics and seasonal forcing in the transmission rates. We investigate the effects of human movement and seasonality on the two-patch dengue transmission dynamics. Motivated by the recent Peruvian dengue data in jungle/rural areas and coast/urban areas, our model mimics the seasonal patterns of dengue outbreaks in two patches. The roles of seasonality and residence-time configurations are highlighted in terms of the seasonal reproduction number and cumulative incidence. Moreover, optimal control theory is employed to identify and evaluate patch-specific control measures aimed at reducing dengue prevalence in the presence of seasonality. Our findings demonstrate that optimal patch-specific control strategies are sensitive to seasonality and residence-time scenarios. Targeting only the jungle (or endemic) is as effective as controlling both patches under weak coupling or symmetric mobility. However, focusing on intervention for the city (or high density areas) turns out to be optimal when two patches are strongly coupled with asymmetric mobility.

## Introduction

Dengue is one of the most important vector-borne diseases, affecting more than 50 million people all around the world [1, 2]. Each year, there are 2.5 billion individuals at risk, including approximately 500,000 severe cases and 22,000 deaths, mostly involving children [3]. *A. aegypti* is the main vector that transmits dengue and it carries four different virus serotypes of the genus Flavivirus. Dengue fever is a mild disease; however, prior strain-specific infections may progress to increased susceptibility to severe dengue hemorrhagic fever and dengue shock syndrome [4, 5]. Despite intensive vector control programs, many countries have experienced dengue reemergence over the last few decades [6, 7]. In 2015, the first dengue vaccine was used in Mexico, however, the effectiveness of the dengue vaccine must be further investigated and assessed [8, 9].

Modeling vector-borne diseases is a challenge for many researchers due to complex factors, including the interplay between vector and host dynamics, spatial or multi-strain, immunity levels, or vaccination [10, 11]. Mathematical modeling has evolved from simpler models to more complex models that include climate changes, socio-economic changes, urbanization and transportation [12, 13]. Particularly, geographic heterogeneity and climate change are some of the key factors when modeling recurrent vector-borne diseases. The spatio-temporal dynamics of infectious disease has been studied using partial differential equations and meta-population models [14, 15]. Human movement plays a significant role on disease reemergence and persistence [16–18]. There are several approaches to model the effect of human movement [17] (references are therein). In that work, two different frameworks have been employed: the Lagrangian framework mimics human commuting behavior, and the Eulerian framework mimics human migration. A discrete-time multi-patch model was used to study the transmission dynamics of dengue in multi-locations by incorporating the movement of people between villages and a city [19]. The impact of commuters between patches are well studied in star networks of villages and a city [20], and quantifying the impact of human mobility on the spread of malaria has also been studied [21]. In these studies, the role of human movement was highlighted in the vector-born disease spread in multi-locations.

Recurrent dengue outbreaks have been commonly observed in South America and other areas of the world [12, 22–24]. Many of these regions have shown seasonal patterns that directly influence the dynamics of dengue transmission [25]. A number of mathematical models have been developed to understand the complexity of dengue transmission dynamics [11, 26]. It was shown that seasonality plays a major role in the size of the mosquito population, which influences the decision of effective strategies to control the disease [27, 28]. Several entomological factors are included in the temperature-dependent entomological parameters such as mortality rates and oviposition rates in a mosquito life-cycle [10]. Recently, ten Bosch et al carried out extensive studies for the dengue transmission dynamics with multiple strains and seasonality [29]. They proposed six models by employing a pattern oriented modeling approach and identified the parameter space so that all proposed models reproduced the observed patterns of dengue outbreaks. Furthermore, they demonstrated that seasonal forcing played a key role in their model selection. These studies highlight the importance of seasonality in the dynamics of dengue transmission.

Therefore, it is critical to incorporate the environmental and seasonal effects into dengue transmission modeling. As reported in previous research [30], the 2000 dengue outbreaks in Peru were examined by using a two-patch model where the jungle areas were always endemic, observing how human movement caused epidemics in the coastal cities. Based on the work in [30], the transmission dynamics of dengue was investigated in a two-patch model [31]. Two patches represented two interconnected locations (a jungle/rural area and a city/urban area) and they were coupled by a residence-time matrix assuming constant transmission rates in both the host and vector populations. The focus was on the overall transmission dynamics between patches under different residence-time configurations for a short-time scale (less than a year). In the present work, we formulate a non-autonomous system to investigate a relatively longer-term dengue dynamics by incorporating seasonality into the two-patch system. The effect of seasonality on patch-specific dynamics including the seasonal basic reproduction number and cumulative incidence is highlighted under various scenarios [32]. We formulate an optimal control problem in a host-vector population in two-patches. The goal is to identify patch-specific strategies that minimize the total number of infected humans and vectors in the presence of seasonality. The effects of seasonality and residence-time configurations are explored on patch-specific preventive controls for a longer time scale.

## Methods

### Two-patch dengue transmission model with seasonality

A two-patch dengue transmission model has been developed for a short time scale (less than one year) in the absence of seasonality [31]. Since recurrent dengue outbreaks occur in many tropical and sub-tropical countries [33], it is more realistic to consider seasonality factors for the long-term dynamics of dengue transmission. For each patch, we add demographics to the host population and seasonality to the vector population and the transmission rates, as proposed in our baseline model [30]. The variables of the vectors are *S*_*v*_ for the susceptible class, *E*_*v*_ for the exposed class and *I*_*v*_ for the infected class, with the total vector population *N*_*v*_ ≡ *S*_*v*_ + *E*_*v*_ + *I*_*v*_. The variables for the human classes are *S*_*h*_ for the susceptible class, *E*_*h*_ for the exposed class, *I*_*h*_ for the infected class, and *R*_*h*_ for the recovered class, with *N*_*h*_ ≡ *S*_*h*_ + *E*_*h*_ + *I*_*h*_ + *R*_*h*_. Since the dengue fatality rate is below 1% under proper medical cares [34], deaths due to dengue are assumed to be negligible, that is, the total human population is assumed to be constant. However, seasonality evidently affects the total population size of the vectors [32, 35–37]. Hence, the seasonality of the vector population is modeled by a periodic vector birth rate.

The two patches are coupled via a residence-time matrix *P* = (*p*_*ij*_)_2×2_ for *i*, *j* = 1, 2. It is assumed that the human residence-time matrix is not affected by seasonality, so *p*_*ij*_ is constant in [0, 1] satisfying the condition $\sum_{j=1}^{2}p_{ij}=1$ for *i* = 1, 2. The residence-time matrix can model the virtual movement of humans between/within patches. More precisely, the coupling parameter *p*_*ij*_ represents the proportion of time that a person residing in patch *i* visits patch *j*. Hence, our model is based on the Lagrangian framework that mimics human commuting behavior [17]. It is assumed that vectors *do not move*, that is, only humans can move across patches. More details of the residence-time matrix are found in the previous work [31]. The two-patch dengue transmission dynamics is captured by the following patch-specific system of nonlinear ordinary differential equations:

$$\begin{array}{ccc} {\dot{S}}_{v1} & = & \mu_{v1}\left(t\right)N_{v1}-\beta_{v1}\left(t\right)\left(p_{11}I_{h1}/N_{h1}+p_{21}I_{h2}/N_{h2}\right)S_{v1}-\mu_{v}S_{v1}\\ {\dot{E}}_{v1} & = & \beta_{v1}\left(t\right)\left(p_{11}I_{h1}/N_{h1}+p_{21}I_{h2}/N_{h2}\right)S_{v1}-\mu_{v}E_{v1}-\kappa E_{v1}\\ {\dot{I}}_{v1} & = & \kappa E_{v1}-\mu_{v}I_{v1}\\ {\dot{S}}_{h1} & = & \mu_{h}N_{h1}-S_{h1}\left(\beta_{h1}\left(t\right)p_{11}I_{v1}/N_{v1}+\beta_{h2}p_{12}I_{v2}/N_{v2}\right)-\mu_{h}S_{h1}\\ {\dot{E}}_{h1} & = & S_{h1}\left(\beta_{h1}\left(t\right)p_{11}I_{v1}/N_{v1}+\beta_{h2}p_{12}I_{v2}/N_{v2}\right)-\gamma E_{h1}-\mu_{h}E_{h1}\\ {\dot{I}}_{h1} & = & \gamma E_{h1}-\delta I_{h1}-\mu_{h}I_{h1}\\ {\dot{R}}_{h1} & = & \delta I_{h1}-\mu_{h}R_{h1}\\ {\dot{S}}_{v2} & = & \mu_{v2}\left(t\right)N_{v2}-\beta_{v2}\left(t\right)\left(p_{12}I_{h1}/N_{h1}+p_{22}I_{h2}/N_{h2}\right)S_{v2}-\mu_{v}S_{v2}\\ {\dot{E}}_{v2} & = & \beta_{v2}\left(t\right)\left(p_{12}I_{h1}//N_{h1}+p_{22}I_{h2}/N_{h2}\right)S_{v2}-\mu_{v}E_{v2}-\kappa E_{v2}\\ {\dot{I}}_{v2} & = & \kappa E_{v2}-\mu_{v}I_{v2}\\ {\dot{S}}_{h2} & = & \mu_{h}N_{h2}-S_{h2}\left(\beta_{h1}\left(t\right)p_{21}I_{v1}/N_{v1}+\beta_{h2}\left(t\right)p_{22}I_{v2}/N_{v2}\right)-\mu_{h}S_{h2}\\ {\dot{E}}_{h2} & = & S_{h2}\left(\beta_{h1}\left(t\right)p_{21}I_{v1}/N_{v1}+\beta_{h2}\left(t\right)p_{22}I_{v2}/N_{v2}\right)-\gamma E_{h2}-\mu_{h}E_{h2}\\ {\dot{I}}_{h2} & = & \gamma E_{h2}-\delta I_{h2}-\mu_{h}I_{h2}\\ {\dot{R}}_{h2} & = & \delta I_{h2}-\mu_{h}R_{h2}, \end{array}$$ (1)

where

$$\begin{array}{c} \begin{array}{c} \mu_{v1}\left(t\right)=\mu_{v}\left(1-\varepsilon_{1}\sin\left(\frac{2\pi t}{365}\right)\right),\;\mu_{v2}\left(t\right)=\mu_{v}\left(1-\varepsilon_{2}\sin\left(\frac{2\pi t}{365}\right)\right). \end{array} \end{array}$$ (2)

The vector birth rate, *μ*_*vi*_ is modeled as a sinusoidal function with a distinct amplitude, which results in the varying vector population size over time (high in the summer and low in the winter) [32, 37, 38]. Dengue is transmitted through two types of interactions: from hosts to vectors and from vectors to hosts. Susceptible vectors acquire infections through contact with infected human individuals at the per-infective and per-capita rate *β*_*vi*_(*t*). It is also transmitted when susceptible individuals are infected via contacts with infected vectors at the per-infective and per-capita rate *β*_*hi*_(*t*).

**Fig 1:**
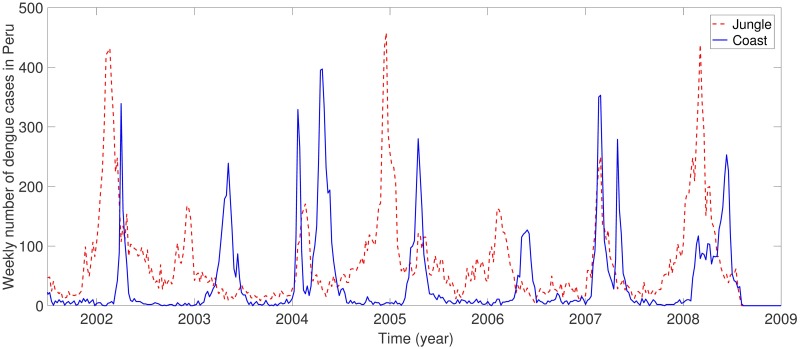
Weekly number of dengue cases in the jungle and the coast in Peru.

The dengue data in Peru from 2001 to 2008 shows recurrent seasonal patterns in Fig 1 (dengue cases in the jungle and the coastal city are shown [25]). Dengue is endemic in the jungle, which is presumed as the driving force of dengue in Peru. In coastal cities, the number of dengue cases are very small in the winter due to the low vector population. Hence, we consider the following two different types of transmission rates, *β*_*vi*_(*t*) (*i* = 1, 2) motivated by the work [30].

(*S*_1_) Seasonality scenario I
  For Patch 1, *β*_*v*1_(*t*) is assumed to be a constant function to model an endemic situation in Patch 1. For Patch 2, the transmission rate has a positive value in the summer, describing that vectors are active and contact hosts, and it is almost zero in the winter, resulting in no incidence rates between hosts and vectors. Thus, *β*_*v*2_(*t*) is modeled as a square wave function, which is frequently used for seasonally varying epidemic models [39, 40].
  Patch-specific transmission rate functions are given by
  $$\begin{array}{c} \begin{array}{c} \beta_{v1}\left(t\right)=0.21,\\ \beta_{v2}\left(t\right)=\left\{\begin{array}{c} 0.21,\;\text{if}\;t\in \left(365\left(n-\frac{1}{4}\right),365\left(n+\frac{1}{4}\right)\right)\;\text{for}\;n=0,1,2,\cdots ,\\ 0.01,\;\text{otherwise.} \end{array}\right. \end{array} \end{array}$$ (3)
(*S*_2_) Seasonality scenario II
  In both patches, *β*_*v*1_(*t*) and *β*_*v*2_(*t*) are sinusoidal type functions with two distinct amplitudes (higher in Patch 1 than Patch 2). The transmission rate in Patch 1 oscillates throughout the year but never goes to zero, in contrast to the one in Patch 2, which is almost zero in the winter [32, 40–42]. atch-specific transmission rate functions are given by
  $$\begin{array}{c} \begin{array}{c} \beta_{v1}\left(t\right)=0.07\left(\cos\frac{2\pi t}{365}+1\right)+0.1,\\ \beta_{v2}\left(t\right)=\left\{\begin{array}{c} 0.21\cos\frac{2\pi t}{365}+0.04,\;\text{if}\;t\in \left(365\left(n-\frac{1}{4}\right),365\left(n+\frac{1}{4}\right)\right)\;\text{for}\;n=0,1,2,\cdots ,\\ 0.01,\;\text{otherwise.} \end{array}\right. \end{array} \end{array}$$ (4)

Also, the patch-specific transmission rates from vectors to hosts are defined as *β*_*hi*_(*t*) = *m*_*i*_(*t*)*β*_*vi*_(*t*), where *m*_*i*_(*t*) = *N*_*vi*_(*t*)/*N*_*hi*_ is the ratio of vector to host [43, 44]. It is assumed that the average vector population size is approximately twice of the human population size ($\frac{N_{vi}}{N_{hi}}\approx 2$) [45].

Note that these parameters (*μ*_*vi*_(*t*), *β*_*vi*_(*t*), *β*_*hi*_(*t*)) are seasonally varying time-dependent (see Fig A in S1 Appendix). The annual oscillations in dengue incidence are caused by complex factors including the seasonality of the vector population and the transmission rates. It is well known that temperature and precipitation play a key role in the seasonal patterns of dengue incidence [32, 46–48]. In general, these parameters increase as temperature or precipitation increases to a certain extent. In our work, we assumed that temperature and precipitation in a jungle/rural area (one patch) are greater than a city/urban area (the other patch). Also, this holds for any two locations with one higher than the other. It has been studied in the previous work when the climate in two locations is similar [31].

The system of nonlinear ordinary differential equations is non-autonomous with periodic forcing terms *μ*_*vi*_(*t*), *β*_*vi*_(*t*) and *β*_*hi*_(*t*) for a 1-year period. Numerical simulations show that the system has an asymptotically stable limit cycle, which is a forced-period solution of period 1-year (See Fig B in S1 Appendix). Since recurrent epidemics are considered here, the focus is on the dynamics of periodic solutions, which is independent of the initial values. Descriptions of the parameters are given in Tables 1 and 2.

**Table 1:** Parameter values in a residence-time matrix.

Coupling intensity	Weak coupling	Strong coupling
Symmetric coupling	*p*_11_ = 0.99 *p*_12_ = 0.01 *p*_21_ = 0.01 *p*_22_ = 0.99	*p*_11_ = 0.7 *p*_12_ = 0.3 *p*_21_ = 0.3 *p*_22_ = 0.7
Asymmetric coupling	*p*_11_ = 0.9 *p*_12_ = 0.1 *p*_21_ = 0.001 *p*_22_ = 0.999	*p*_11_ = 0.7 *p*_12_ = 0.3 *p*_21_ = 0.001 *p*_22_ = 0.999

**Table 2:** Definitions and baseline values of parameters used in numerical simulations.

Parameters	Description	Value	Ref
*p*_*ij*_	Proportion of time for hosts visiting patch *j* from patch *i*	0–1	[31]
*γ*	Progression rate from latent to infectious for host (days^−1^)	1/5.5	[45]
*κ*	Progression rate from latent to infectious for vector (days^−1^)	1/12	[45]
*δ*	Recovery rate (days^−1^) for infectious class for host (days^−1^)	1/5.0	[45]
*ε*_1_	Amplitude of oscillations in vector birth rate in Patch 1	0, 0.1	[49–51]
*ε*_2_	Amplitude of oscillations in vector birth rate in Patch 2	0.2	[49–51]
*μ*_*h*_	Host birth/death rate (days^−1^)	1/(65 * 365)	[45]
*μ*_*v*_	Average vector birth/death rate (days^−1^)	1/14	[45]
*N*_*hi*_	Total number of hosts in patch *i*	100000	Assumption
*N*_*vi*_	Total number of vectors in patch *i*	**	[45]
*μ*_*vi*_	Vector birth rate in patch *i* (days^−1^)	**	[49–51]
*m*_*i*_	Number of vectors per host in patch *i*	**	[43, 44]
*β*_*vi*_	Transmission rate from host to vector in patch *i* (days^−1^)	**	[43]
*β*_*hi*_	Transmission rate from vector to host in patch *i* (days^−1^)	**	[43, 44]
*b*	The upper bound of control (efficient reduction rates, days^−1^)	0.35	
*W*_*i*_	Weight constants on controls *i* = 3, 4	5000, 10000, 50000	

(** denotes the time-dependent parameters)

### Optimal controls in two-patch dengue transmission model

More recently, optimal control theory has been successfully employed in many biological and epidemiological models [52–54]. Optimal control problems have been formulated to identify optimal strategies and study the impact of control measures for vector-borne diseases [55, 56]. In this section, we formulate an optimal control problem in order to implement effective patch-specific control measures that take into account different coupling and seasonality cases. The two-patch dengue model is modified by incorporating the patch-specific control functions (1 − *u*_*i*_(*t*)) into the incidence rates at which humans and vectors get infected for patch *i* (*i* = 1, 2) in Eq (1). Preventive control efforts may involve the application of pesticide (sprays), mosquito repellents, reduction of the impact of vector breeding grounds, or the results of education campaigns, which increase personal protection. It is assumed that these preventive interventions do not reduce the total vector population significantly, and the effect of these interventions implicitly translates in reductions of transmission between vectors and hosts per unit time. In particular, incidence rates including controls are modified as $\beta_{vi}\left(1-u_{i}\left(t\right)\right)S_{vi}\sum_{j=1}^{2}p_{ji}I_{hj}$ and $S_{hi}\sum_{j=1}^{2}\beta_{hj}\left(1-u_{j}\left(t\right)\right)p_{ij}I_{vj}$ for *i* = 1, 2. The controlled two-patch system is given as

$$\begin{array}{ccc} {\dot{S}}_{v1} & = & \mu_{v1}\left(t\right)N_{v1}-\beta_{v1}\left(t\right)\left(1-u_{1}\left(t\right)\right)\left(p_{11}I_{h1}/N_{h1}+p_{21}I_{h2}/N_{h2}\right)S_{v1}-\mu_{v}S_{v1}\\ {\dot{E}}_{v1} & = & \beta_{v1}\left(t\right)\left(1-u_{1}\left(t\right)\right)\left(p_{11}I_{h1}/N_{h1}+p_{21}I_{h2}/N_{h2}\right)S_{v1}-\mu_{v}E_{v1}-\kappa E_{v1}\\ {\dot{I}}_{v1} & = & \kappa E_{v1}-\mu_{v}I_{v1}\\ {\dot{S}}_{h1} & = & \mu_{h}N_{h1}-S_{h1}\left(\beta_{h1}\left(t\right)\left(1-u_{1}\left(t\right)\right)p_{11}I_{v1}/N_{v1}+\beta_{h2}\left(t\right)\left(1-u_{2}\left(t\right)\right)p_{12}I_{v2}/N_{v2}\right)-\mu_{h}S_{h1}\\ {\dot{E}}_{h1} & = & S_{h1}\left(\beta_{h1}\left(t\right)\left(1-u_{1}\left(t\right)\right)p_{11}I_{v1}/N_{v1}+\beta_{h2}\left(t\right)\left(1-u_{2}\left(t\right)\right)p_{12}I_{v2}/N_{v2}\right)-\gamma E_{h1}-\mu_{h}E_{h1}\\ {\dot{I}}_{h1} & = & \gamma E_{h1}-\delta I_{h1}-\mu_{h}I_{h1}\\ {\dot{R}}_{h1} & = & \delta I_{h1}-\mu_{h}R_{h1}\\ {\dot{S}}_{v2} & = & \mu_{v2}\left(t\right)N_{v2}-\beta_{v2}\left(t\right)\left(1-u_{2}\left(t\right)\right)\left(p_{12}I_{h1}/N_{h1}+p_{22}I_{h2}/N_{h2}\right)S_{v2}-\mu_{v}S_{v2}\\ {\dot{E}}_{v2} & = & \beta_{v2}\left(t\right)\left(1-u_{2}\left(t\right)\right)\left(p_{12}I_{h1}//N_{h1}+p_{22}I_{h2}/N_{h2}\right)S_{v2}-\mu_{v}E_{v2}-\kappa E_{v2}\\ {\dot{I}}_{v2} & = & \kappa E_{v2}-\mu_{v}I_{v2}\\ {\dot{S}}_{h2} & = & \mu_{h}N_{h2}-S_{h2}\left(\beta_{h1}\left(t\right)\left(1-u_{1}\left(t\right)\right)p_{21}I_{v1}/N_{v1}+\beta_{h2}\left(t\right)\left(1-u_{2}\left(t\right)\right)p_{22}I_{v2}/N_{v2}\right)-\mu_{h}S_{h2}\\ {\dot{E}}_{h2} & = & S_{h2}\left(\beta_{h1}\left(t\right)\left(1-u_{1}\left(t\right)\right)p_{21}I_{v1}/N_{v1}+\beta_{h2}\left(t\right)\left(1-u_{2}\left(t\right)\right)p_{22}I_{v2}/N_{v2}\right)-\gamma E_{h2}-\mu_{h}E_{h2}\\ {\dot{I}}_{h2} & = & \gamma E_{h2}-\delta I_{h2}-\mu_{h}I_{h2}\\ {\dot{R}}_{h2} & = & \delta I_{h2}-\mu_{h}R_{h2}. \end{array}$$ (5)

Patch-specific optimal controls are obtained by minimizing the number of both infected hosts and vectors and the cost of implementation strategies over a finite time interval. The objective functional to be minimized is defined as

$$\begin{array}{c} J\left(u_{1}\left(t\right),u_{2}\left(t\right)\right)=\int_{0}^{t_{f}}W_{1}\left(I_{h1}\left(t\right)+I_{v1}\left(t\right)\right)+W_{2}\left(I_{h2}\left(t\right)+I_{v2}\left(t\right)\right)+\frac{1}{2}W_{3}u_{1}^{2}\left(t\right)+\frac{1}{2}W_{4}u_{2}^{2}\left(t\right)dt, \end{array}$$ (6)

where *W*_1_ and *W*_2_ are weight constants on the infected hosts and vectors for Patch 1 and Patch 2, respectively. Weight constants, *W*_3_ and *W*_4_ are the relative costs of the implementation of the preventive controls for Patch 1 and Patch 2, respectively. We model the control efforts as a linear combination of quadratic terms, $u_{i}^{2}\left(t\right)$ (*i* = 1, 2) due to the convexity of the controls in the objective functional. Then, we seek an optimal pair (U*, X*) such that

$$\begin{array}{c} J\left(U^{*}\right)=min\left\{J\left(U\right)|U\in \Omega \right\}, \end{array}$$ (7)

where Ω = {(*u*_1_(*t*), *u*_2_(*t*)) ∈ (*L*^1^(0, *t*_*f*_))^2^ ∥ *a* ≤ *u*_*i*_(*t*) ≤ *b*, *t* ∈ [0, *t*_*f*_], *i* = 1, 2} subject to the state system Eq (5) with *X* = (*S*_*v*1_, *E*_*v*1_, *I*_*v*1_, *S*_*h*1_, *E*_*h*1_, *I*_*h*1_, *R*_*h*1_, *S*_*v*2_, *E*_*v*2_, *I*_*v*2_, *S*_*h*2_, *E*_*h*2_, *I*_*h*2_, *R*_*h*2_) and *U* = (*u*_1_, *u*_2_). The existence of optimal controls is guaranteed from standard results on optimal control theory [57]. Pontryagin’s Maximum Principle is used to establish necessary conditions that must be satisfied by an optimal solution [58]. Derivations of the necessary conditions are shown in Section C in S1 Appendix.

## Simulation results

In this section, we present the two-patch dengue transmission dynamics in the absence of controls and in the presence of controls. The roles of a residence-time matrix and seasonality are investigated. More specifically, the residence-time matrix configurations include the coupling intensity and the mobility patterns. For instance, weak coupling implies that most humans stay in their own patch while strong coupling implies that certain proportions of humans visit the other patch. Mobility patterns represent the symmetry of human movement between the two patches. For example, if the proportion of humans visiting from patch *i* to patch *j* is the same as for patch *j* to patch *i*, then it is symmetric. Asymmetric mobility implies that the time spent for each proportion becomes more asymmetric. For the asymmetric mobility pattern, we assume that more humans from the jungle/rural area (Patch 1) visit the city/urban area (Patch 2) than the other way around.

We first focus on the case when the two patches have the similar population size (*N*_*h*1_ = *N*_*h*2_). Then, we will discuss the impact of different subpopulation sizes later. The population size of the coastal area is about twice as the population size of the jungle area in Peru [59], hence we consider the second case of *N*_*h*1_ < *N*_*h*2_ with *N*_*h*2_ = 2*N*_*h*1_.

### Two-patch dengue transmission dynamics in the absence of controls

#### The seasonal reproduction number

The basic reproduction number $R_{0}$ is the average number of secondary infectious cases when one infectious individual is introduced into a whole susceptible population. It can be calculated as the dominant eigenvalue of the next generation matrix for an autonomous system [60]. However, due to several time-dependent parameters in our system, the basic reproduction number varies in time. Therefore, the seasonal reproduction number $R_{s}$ is computed by the same procedure as for time-dependent parameters [32, 61].

$$\begin{array}{c} R_{s}=\sqrt{\frac{\kappa \gamma }{2C}\left(\phi_{1}+\sqrt{\phi_{2}}\right)} \end{array}$$ (8)

where

$$\begin{array}{c} \phi_{1}=B_{1}+B_{2}\\ \phi_{2}=B_{3}\left(A_{1}+A_{2}+A_{3}\right). \end{array}$$

$$\begin{array}{ccc} C & = & \mu_{v}\;\left(\kappa +\mu_{v}\right)\;\left(\gamma +\mu_{h}\right)\;\left(\delta +\mu_{h}\right)\\ B_{1} & = & \left(\beta_{h1}\beta_{v1}p_{11}^{2}S_{h1}S_{v1}\right)/\left(N_{h1}N_{v1}\right)+\left(\beta_{h1}\beta_{v1}p_{21}^{2}S_{h2}S_{v1}\right)/\left(N_{h2}N_{v1}\right)\\ B_{2} & = & \left(\beta_{h2}\beta_{v2}p_{12}^{2}S_{h1}S_{v2}\right)/\left(N_{h1}N_{v2}\right)+\left(\beta_{h2}\beta_{v2}p_{22}^{2}S_{h2}S_{v2}\right)/\left(N_{h2}N_{v2}\right)\\ B_{3} & = & 1/(N_{h1}^{2}N_{h2}^{2}N_{v1}^{2}N_{v2}^{2})\\ A_{1} & = & \beta_{h1}^{2}\beta_{v1}^{2}N_{v2}^{2}{\left(N_{h2}p_{11}^{2}S_{h1}+N_{h1}p_{21}^{2}S_{h2}\right)}^{2}S_{v1}^{2}\\ A_{2} & = & 2\beta_{h1}\beta_{h2}\beta_{v1}\beta_{v2}N_{v1}N_{v2}(N_{h2}^{2}p_{11}^{2}p_{12}^{2}S_{h1}^{2}+N_{h1}N_{h2}\left(4p_{11}p_{12}p_{21}p_{22}-p_{12}^{2}p_{21}^{2}-p_{11}^{2}p_{22}^{2}\right)S_{h1}S_{h2}\\ & + & N_{h1}^{2}p_{21}^{2}p_{22}^{2}S_{h2}^{2})S_{v1}S_{v2}\\ A_{3} & = & \beta_{h2}^{2}\beta_{v2}^{2}N_{v1}^{2}{\left(N_{h2}p_{12}^{2}S_{h1}+N_{h1}p_{22}^{2}S_{h2}\right)}^{2}S_{v2}^{2} \end{array}$$

More details of the calculation of $R_{s}$ are given in Section B in S1 Appendix. In Fig C in S1 Appendix, the time series of the global seasonal reproduction number $R_{s}$ and human incidence are displayed for symmetric coupling. The incidence of Patch 2 increases when $R_{s}>1$ and decreases when $R_{s}<1$. The time series of $R_{s}$ follows *β*_*v*2_(*t*) rather than *β*_*v*1_(*t*). This implies that the global seasonal reproduction number is more sensitive to *β*_*v*2_(*t*).

#### The effects of residence-time matrix configurations and seasonality scenarios

**Fig 2:**
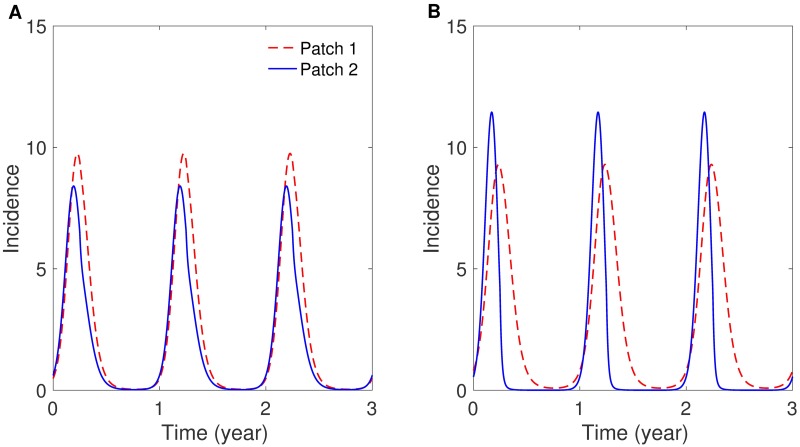
The effect of coupling strength on patch-specific incidence. (A) Strong coupling (*p*_12_ = *p*_21_ = 0.3) and (B) weak coupling (*p*_12_ = *p*_21_ = 0.01) for the case of symmetric coupling and the sinusoidal type seasonality (*S*_2_).

**Fig 3:**
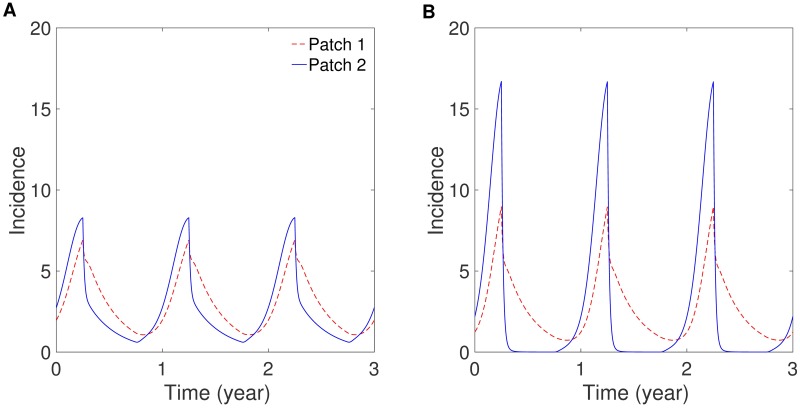
The effect of symmetry of movement on patch-specific incidence. (A) Symmetric coupling (*p*_12_ = *p*_21_ = 0.3) and (B) asymmetric coupling (*p*_12_ = 0.3 and *p*_21_ = 0.001) for the case of strong coupling and the square-wave type seasonality (*S*_1_).

First, the impacts of a residence-time matrix and seasonality scenarios are presented on patch-specific incidences. Fig 2 shows the results under different coupling intensities using symmetric mobility: weakly coupled (left panel) and strongly coupled (right panel). As coupling becomes stronger, the peak sizes of both patches become more similar. This can be interpreted as the population from different patches being well mixed, and so the dynamics of each patch become similar. Fig 3 presents the effects of mobility patterns: *symmetric coupling* (*p*_12_ = *p*_21_ on the left) and *asymmetric coupling* (*p*_12_ > *p*_21_ on the right). The peak sizes in both patches become higher as the coupling becomes more asymmetric, that is, the proportion of humans visiting the city from the jungle becomes larger. The effect of seasonality scenarios is presented in Fig D in S1 Appendix using symmetric mobility. This figure shows slightly stronger synchronization between two patches under the sinusoidal type scenario (*S*_2_) than for under the square wave type scenario (*S*_1_). The qualitative behaviors in both patches are not significantly sensitive to the seasonality scenario.

#### Cumulative incidence

**Fig 4:**
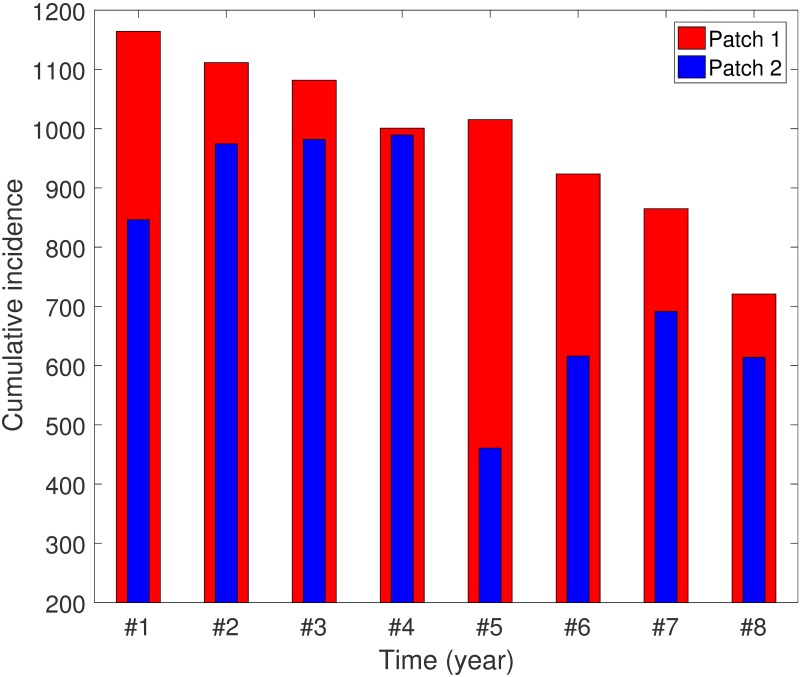
The effect of seasonality and coupling scenarios on patch-specific cumulative incidence for one year. #1–#4: Square-wave type seasonality (*S*_1_), #5–#8: Sinusoidal type seasonality (*S*_2_); #1, #5: *p*_12_ = 0.01, *p*_21_ = 0.01 (weak / symmetric); #2, #6: *p*_12_ = 0.1, *p*_21_ = 0.001 (weak / asymmetric); #3, #7: *p*_12_ = 0.3, *p*_21_ = 0.3 (strong / symmetric); #4, #8: *p*_12_ = 0.3, *p*_21_ = 0.001 (strong / asymmetric).

The final epidemic size is one of the most important epidemiological quantities for the standard SIR model with a constant transmission in the absence of the demographic effect [62]. For seasonally varying epidemic models, cumulative incidence (CI) for a finite time interval can be computed instead of the final epidemic size [41, 42]. Throughout our manuscript, the cumulative incidence is computed as $\int_{0}^{365}\kappa E_{hi}\left(t\right)dt$ for a one-year period. We present the effects of various coupling and seasonality scenarios on CI in Fig 4. Cumulative incidence in Patch 1 is always larger than that in Patch 2, even though peak sizes in Patch 2 are higher than for Patch 1 (see Figs 2 and 3). This is due to the fact that the vector population remains constantly high in the endemic region. It is also observed that the dynamics in Patch 2 is more sensitive to residence-time configurations since people tend to move from the jungle to the city (Patch 2 has a higher density).

The impact of different residence-time configurations on cumulative incidence is shown in Fig E of S1 Appendix. Clearly, CI on Patch 1 decreases as *p*_12_ increases in a linear fashion, that is, as more people from Patch 1 visit Patch 2. However, CI on Patch 2 becomes more complex as *p*_12_ increases. For the case when *p*_21_ is small (*p*_21_ ≤ 0.2), CI on Patch 2 increases as *p*_12_ gets larger. On the other hand, when there is more visiting from Patch 2 to Patch 1, then CI on Patch 2 decreases even for a lager value of *p*_12_. Overall CI (Patch 1 + Patch 2) shows a similar tendency as that for CI on Patch 1, that is, an increase in visiting from Patch 1 to Patch 2 makes CI smaller, while the opposite is true for more people visiting from Patch 2 to Patch 1. Moreover, we have carried out sensitivity analysis for seasonality parameters, *ε*_*i*_, *μ*_*v*_, *β*_*v*_ and *β*_*h*_, and the effects of these parameters on CI are shown in Fig I of S1 Appendix.

### Controlled two-patch dengue transmission dynamics

We present the two-patch dengue transmission dynamics in the presence of optimal controls. Numerical solutions to Eq (7) are obtained by the standard scheme (a two point boundary method [52]), which is employed as follows. First, the state system Eq (5) is solved forward in time with initial conditions and an initial guess for the control. Second, the adjoint system with transversality conditions is solved backward in time. Third, the optimality condition is updated. These three steps are iterated until convergence is achieved. There are some critical control parameter values that affect optimal solutions greatly such as weight constants and a simulation time duration. For the weight constants, *W*_1_ = *W*_2_ = 1 and *W*_3_ = *W*_4_ = 10000 are used throughout the simulations and two different simulation time durations (three and six years) are used. Parameter values are given in Tables 1 and 2.

#### The effect of coupling and seasonality scenarios

**Fig 5:**
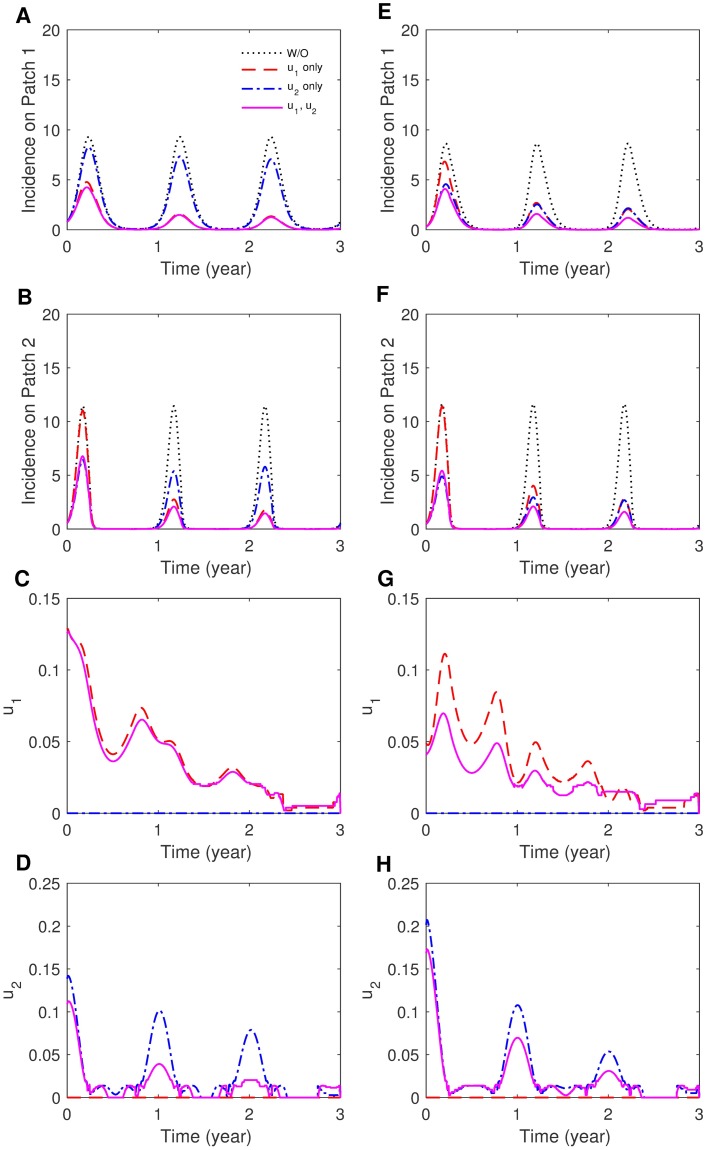
The effect of coupling intensity on patch-specific incidence and optimal control functions. (A)-(D) Weak coupling (*p*_12_ = 0.1, *p*_21_ = 0.001) and (E)-(H) strong coupling (*p*_12_ = 0.3, *p*_21_ = 0.001) for the case of asymmetric coupling and the sinusoidal type seasonality (*S*_2_).

We consider three distinct control strategies: (1) both patches, (2) only Patch 1 and (3) only Patch 2. First, the impact of coupling intensity is explored under the same coupling symmetry and seasonality. Fig 5 shows human incidence and optimal controls for *p*_12_ = 0.1, *p*_21_ = 0.001 (weak) and *p*_12_ = 0.3, *p*_21_ = 0.001 (strong) using *S*_1_ (asymmetric mobility). Obviously, applying control strategies to both patches gives the greatest incidence reduction, but it is worth noting that controlling only Patch 1 is as effective as controlling both patches under weak coupling. This is in contrast to strong coupling, where controlling only Patch 2 is more effective than controlling Patch 1. This can be interpreted such that when mobility between the two patches is relatively small, it is effective to control the endemic region (Patch 1). While the residents in two patches are well mixed due to higher mobility, targeting the region where the population density is higher (Patch 2) is optimal. For symmetric mobility, the results are not significantly sensitive to coupling intensity; for weak or strong coupling, focusing on Patch 1 is as effective as controlling both patches. The effectiveness of the controls becomes higher as coupling is weaker (i.e., most people stay in their own patch).

**Fig 6:**
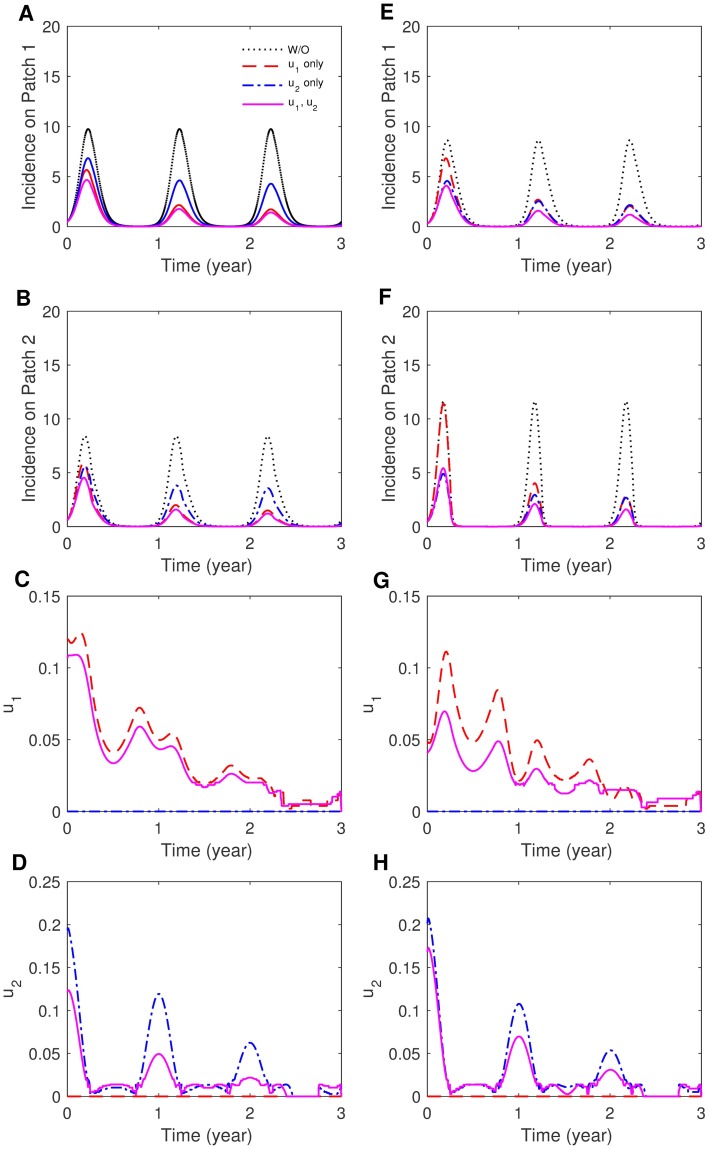
The effect of symmetry of movement on patch-specific incidence and optimal control functions. (A)-(D) Symmetric movement (*p*_12_ = *p*_21_ = 0.3) and (E)-(H) asymmetric movement (*p*_12_ = 0.3, *p*_21_ = 0.001) for the case of strong coupling and the sinusoidal type seasonality (*S*_2_).

The impact of the mobility pattern on the controlled dynamics is investigated in Fig 6. Results are compared for *p*_12_ = 0.3, *p*_21_ = 0.3 (symmetric) and *p*_12_ = 0.3, *p*_21_ = 0.001 (asymmetric) using *S*_2_. Again, controlling both patches shows the greatest incidence reduction in both patches. For symmetric coupling, applying control strategies for only Patch 1 (endemic area) is more effective than controlling for only on Patch 2 due to the fact that there is a higher rate of movement between the patches and almost identical human densities on both patches. For asymmetric coupling, there are more people from Patch 1 visiting Patch 2 than from Patch 2 to Patch 1, hence, controlling only Patch 2 (higher density) is more effective. Next, we compared the results with two distinct seasonality scenarios, *S*_1_ (a square-wave type) and *S*_2_ (a sinusoidal type), under the same coupling strength and symmetry mobility. Fig F in S1 Appendix shows human incidence and controls for (*S*_1_) and (*S*_2_) using *p*_12_ = 0.1 and *p*_21_ = 0.001. Patch 1 is more sensitive to the change of seasonality scenario than Patch 2 due to a bigger change of *β*_*v*1_ than *β*_*v*2_. Our results show that the seasonality scenario does not significantly affect the qualitative behavior of the dynamics. The controlled dynamics is more sensitive to coupling scenarios than the seasonality scenario.

Regardless of all residence-time configurations or seasonality scenarios, in general, (the overall profiles of controls) intensive controls should be given at the first year in both patches. Moreover, due to the endemicity of Patch 1, it turns out that continuous control in a decreasing manner during the entire time duration is necessary in Patch 1. However, for Patch 2, intermittent control is more effective, that is, control should be concentrated during the summer (right before the outbreak) due to seasonal patterns in Patch 2. If there are sufficient resources available, control measures can be applied to both patches, and if resources are not sufficient, it is better to directly target the location of interest. Therefore, under limited resources available, it becomes more critical to take into consideration the residence-time configurations and seasonality scenarios when public health officials make a decision on which area should be targeted.

#### The effects of duration and weight constants

**Fig 7:**
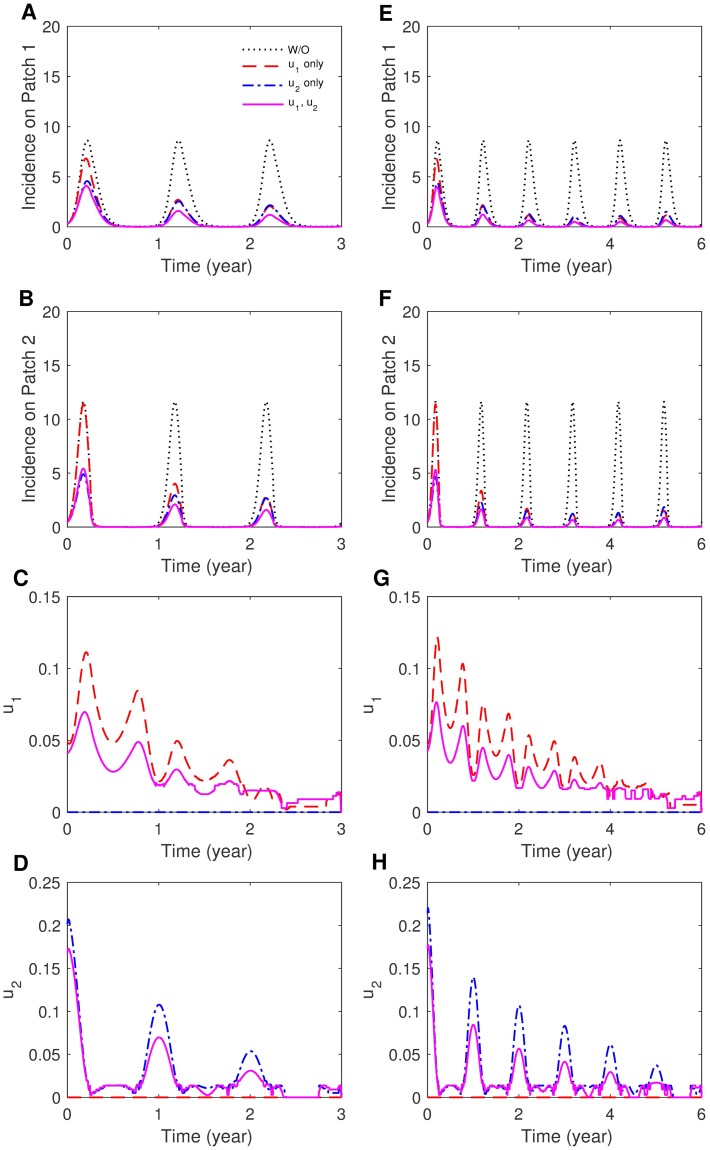
The effect of control duration on patch-specific incidence and optimal control functions. (A)-(D) Duration of three years and (E)-(H) duration of six years for the case of strong and asymmetric coupling (*p*_12_ = 0.3, *p*_21_ = 0.001) with the sinusoidal type seasonality (*S*_2_).

**Fig 8:**
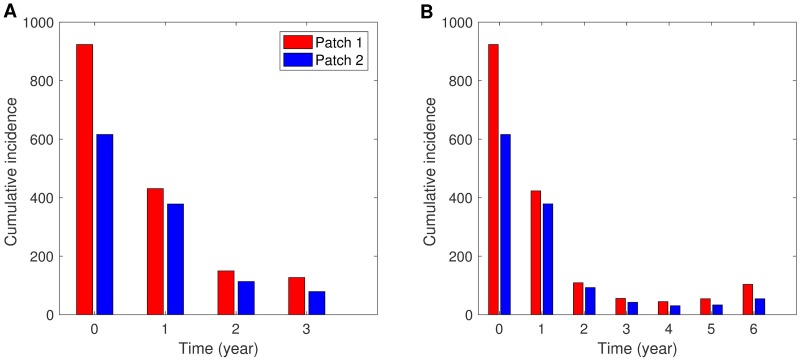
Cumulative incidence for one year under different control duration. When both patches are controlled for (A) three years and (B) six years, cumulative incidence for one year is compared with cumulative incidence for one year without control (displayed on time 0) using *p*_12_ = 0.1, *p*_21_ = 0.001 and the sinusoidal type seasonality (*S*_2_).

In the previous section, all results are computed under a simulation duration of three years. The simulation duration is doubled to six years to investigate the effect of time duration on the two-patch controlled dynamics. Fig 7 displays the human incidence and optimal controls under three years and six years using *p*_12_ = 0.3, *p*_21_ = 0.001. Putting controls in both patches is expected to best reduce the incidences. For the case of limited resources available, controlling only Patch 2 is sufficiently effective to reduce dengue incidence on both patches as we observed in the three year duration case. Next, Fig 8 shows cumulative incidence for each patch, when both patches are controlled for three years and six years, which are compared with CI without control. It is observed that under the duration of six years, cumulative incidence has been reduced significantly. The weight constant can be considered as the relative cost of control implementation, and a larger value represents a relatively higher cost. The impact of control weight constants is illustrated under several values of weight constants. Fig G in S1 Appendix shows human incidence and optimal controls using *W*_3_ = *W*_4_ = 5000, 10000, 50000 (*W*_3_ = *W*_4_ = 10000 is taken as the baseline value, which is used throughout this paper). Obviously, the impact is straightforward; for higher costs, the control decreases, which leads to larger incidences.

#### The impact of different subpopulation sizes

**Fig 9:**
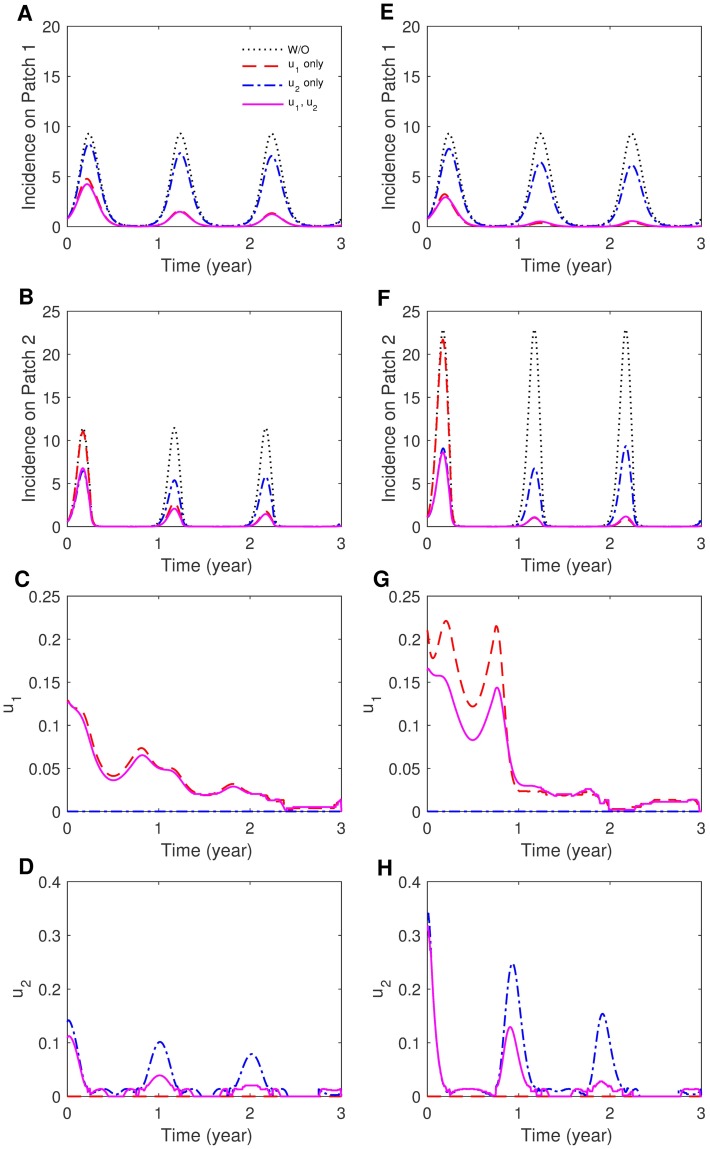
The effect of different patch sizes on patch-specific incidence and optimal controls. (A)-(D) *N*_*h*1_ = *N*_*h*2_ = 10^5^ and (E)-(H) *N*_*h*1_ = 10^5^, *N*_*h*2_ = 2*N*_*h*1_ for the case of asymmetric and weak coupling (*p*_12_ = 0.1, *p*_21_ = 0.001) with the sinusoidal type seasonality (*S*_2_). Control efforts increase in both patches due to the increment of *N*_*h*2_ in (E)-(H). More intensive efforts should be implemented in Patch 2 (H).

In the previous sections, we have focused on the effect of coupling and seasonality scenarios when each patch has the same host population size. Here we have investigated the impact of different patch-sizes of human individuals by comparing the results of two scenarios (Case 1: *N*_*h*1_ = *N*_*h*2_ and Case 2: *N*_*h*1_ < *N*_*h*2_ with *N*_*h*2_ = 2*N*_*h*1_). Patch-specific incidences are illustrated in Fig H in S1 Appendix. As *N*_*h*2_ is doubled, Patch 1 incidence barely changes, but Patch 2 incidence increases almost twice. Patch-specific incidences and controls are presented in Fig 9. As the population size of Patch 2 becomes larger, Patch 2 incidence increases, hence, control efforts increase in both patches. Note that more intensive efforts should be implemented in Patch 2. The effect of coupling scenarios on the results of Case 2 remains qualitatively similar as the ones of Case 1.

## Discussions

We have investigated the dynamics of dengue transmission in a seasonally varying two-patch dengue system. It is assumed that the two patches represent two locations that have a constant and well-defined visiting relationship modeled by a residence-time matrix. Motivated by the recurrent dengue outbreaks in Peru, host demographics and seasonality have been included in the previous model [31]. We assumed that one patch is endemic (jungle/rural areas), and we modeled how human visiting between the two patches caused epidemics in the other patch (coast/urban areas) [30]. The effects of two distinct seasonality scenarios have been investigated under different residence-time configurations. Stronger synchronization occurs for the sinusoidal type transmission rate function than for the square-wave type. Regardless of residence-time configurations or seasonality, the dengue dynamics in both patches become similar as coupling strength becomes stronger. Also, human incidences in both patches have higher peaks as the residence-time matrix becomes more asymmetric regardless of seasonality. The seasonal reproduction number and cumulative incidence are investigated under various scenarios. Overall cumulative incidence increases as coupling intensity becomes stronger for both the symmetric and asymmetric cases. However, overall cumulative incidence decreases as *p*_12_ increases, that is, more people visit the city from the jungle.

We developed an optimal control framework to identify optimal patch-specific control strategies under various scenarios. First, we identify optimal strategies and compare the controlled dynamics with the results in the absence of controls. Our results indicate that, as expected, controlling the two patches simultaneously gives the best reduction in dengue prevalence. However, for the case when only one patch can be controlled due to limited resources, the resulting control strategies become more sensitive to residence-time configurations. For instance, focusing on Patch 1 (the endemic area) is turned out to be optimal under weak coupling or symmetric mobility patterns. However, focusing only on Patch 2 (the city with higher human density) is more effective under strong coupling with asymmetric mobility patterns. Moreover, the results of optimal problems are sensitive to the subpopulation sizes. As the population size of Patch 2 is increased, more intensive control efforts should be implemented in both patches.

Even though our work is motivated by dengue incidence in Peru, we aim to build a general model that can provide an experimental tool for any two interconnected locations with well defined commuting or visiting relationships. Human mobility patterns are one of critical factors for dengue transmission dynamics, however, we rather use a residence-time matrix to capture the effect of virtual human movements due to the lack of real commuting data.

Dengue fever is a challenge for vector control and education program, even with the joint efforts of government and community, and the potential use of partially effective vaccines at the population level. More advanced mathematical modeling should involve vector and host interactions, dynamics of circulating dengue serotypes, and geographical and demographical/behavioral factors for both vector and host. Hence, understanding the mechanism behind the complex spatial-temporal dynamics of dengue disease requires multidisciplinary and transdisciplinary efforts.

Identification and evaluation of optimal strategies that minimize the spread of dengue have been explored through the use of mathematical models. The work presented here can model the dengue transmission dynamics in two seasonally varying locations that are geographically close and similar in population size. Our results show the challenges that public health officials face in how resources should be allocated in heterogeneous environments. Our findings suggest that public health officials should focus on combating dengue in the area with a higher population density (cities) or in the region with a higher transmission rate where dengue is endemic, depending on the residence-time configurations and the amount of available resources.

## Supporting information

S1 Appendix Supplementary figures and appendices. (PDF)
