# Supplementary material for: Assessment of optimal strategies in a two-patch dengue transmission model with seasonality
Source: PLoS One. 2017 Mar 16;12(3):e0173673. doi: 10.1371/journal.pone.0173673 (PMC5354280; doi:10.1371/journal.pone.0173673)
Supplement: S1 Appendix — (PDF) [file pone.0173673.s001.pdf]

Supplementary Figures and Appendices

A. Supplementary Figures

Two distinct seasnonality scenarios

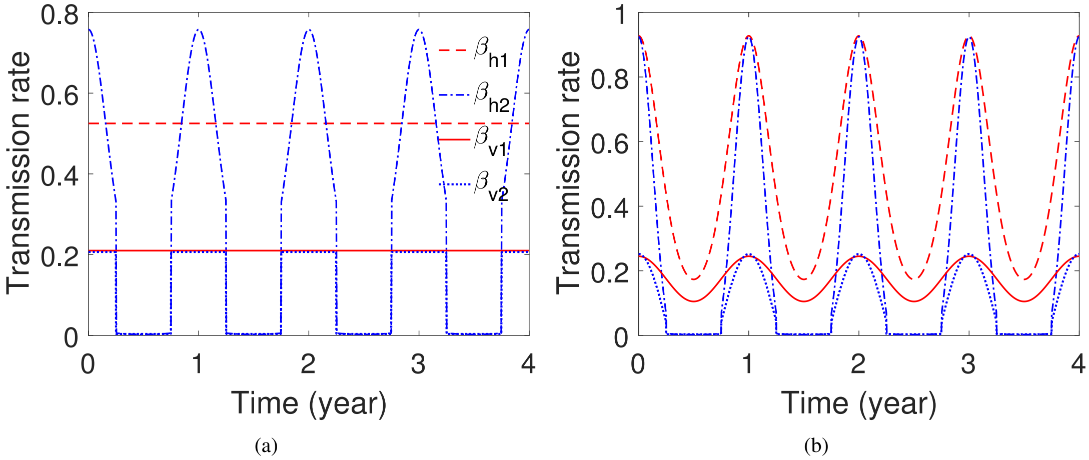

Figure A: Two distinct seasonality scenarios: time-dependent and patch-specific transmission rates for vectors and hosts are displayed (β_{vi} and β_{hi} for $i = 1, 2$) (a) the square wave type (S_1) and (b) the sinusoidal type (S_2). Note that the transmission rates for Patch 1 (jungle areas) show endemicity in both scenarios.

Limit cycle

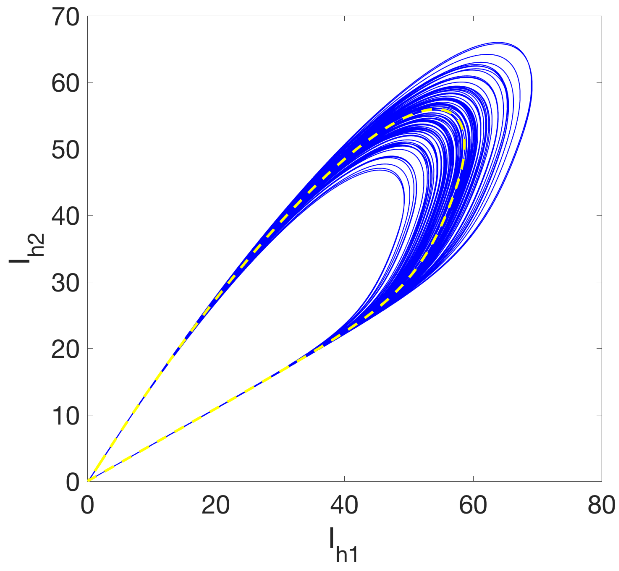

Figure B: There exists the asymptotically stable limit cycle in our two-patch dengue system. Phase plane of patch-specific incidence for human (I_{h1}, I_{h2}) is illustrated using $p_{12} = p_{21} = 0.3$ and the sinusoidal type (S_2): trajectories (blue) and the asymptotically stable limit cycle (yellow dashed) are shown.

The seasonal reproduction number

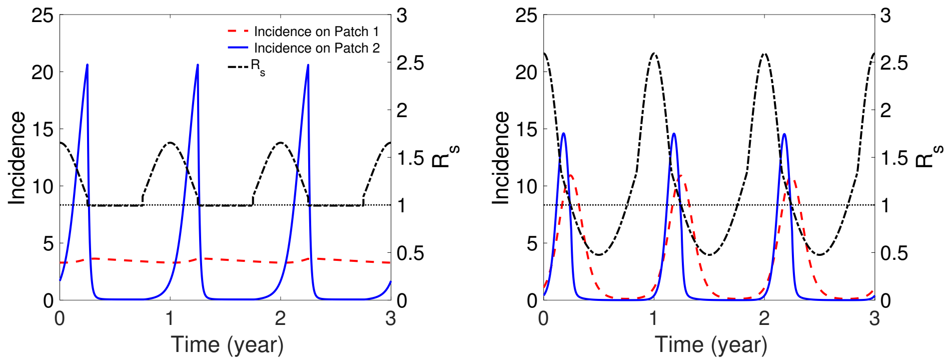

(a) (b)

Figure C: The seasonal reproduction number $\mathcal{R}_s$ (black dash-dotted) and patch-specific human incidence are displayed for Patch 1 (red dashed) and Patch 2 (blue solid) using $p_{12} = p_{21} = 0.01$ (a) the square-wave type (S_1) and (b) the sinusoidal type (S_2). Note that the dotted line represents $\mathcal{R}_s = 1$.

The effects of seasonality scenarios

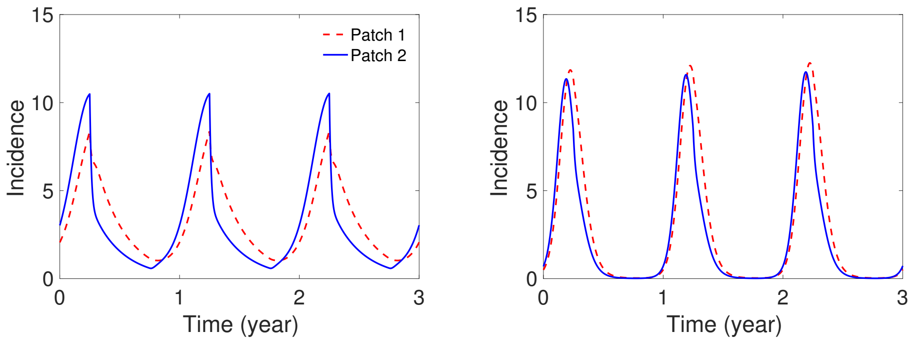

(a) (b)

Figure D: The effects of seasonality types on patch-specific incidence are illustrated under symmetric and strong coupling ($p_{12} = p_{21} = 0.3$) (a) the square-wave type (S_1) and (b) the sinusoidal type (S_2).

The effects of residence-time matrix configurations on cumulative incidence

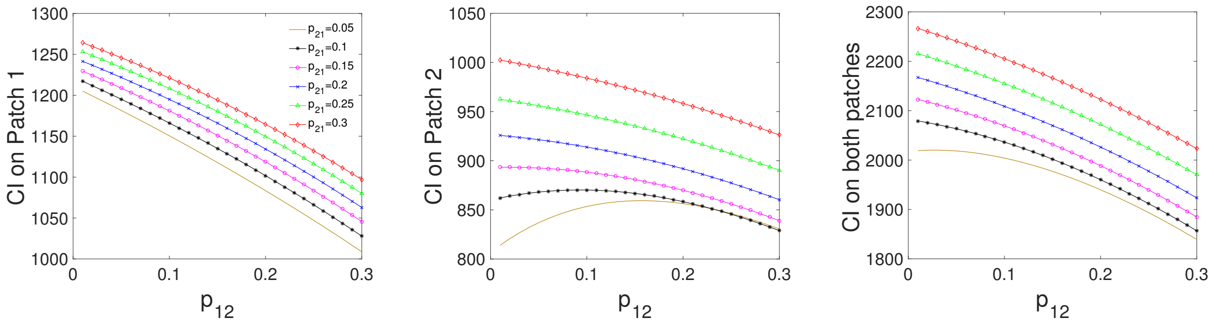

(a) (b) (c)

Figure E: The effects of residence-time matrix configurations on cumulative incidence are shown under the square wave type (S_1) (a) Patch 1, (b) Patch 2 and (c) both patches vs. p_{12} for $p_{21} = 0.05,\ 0.1, \cdots, 0.3$.

Optimal control strategies for two seasonality scenarios

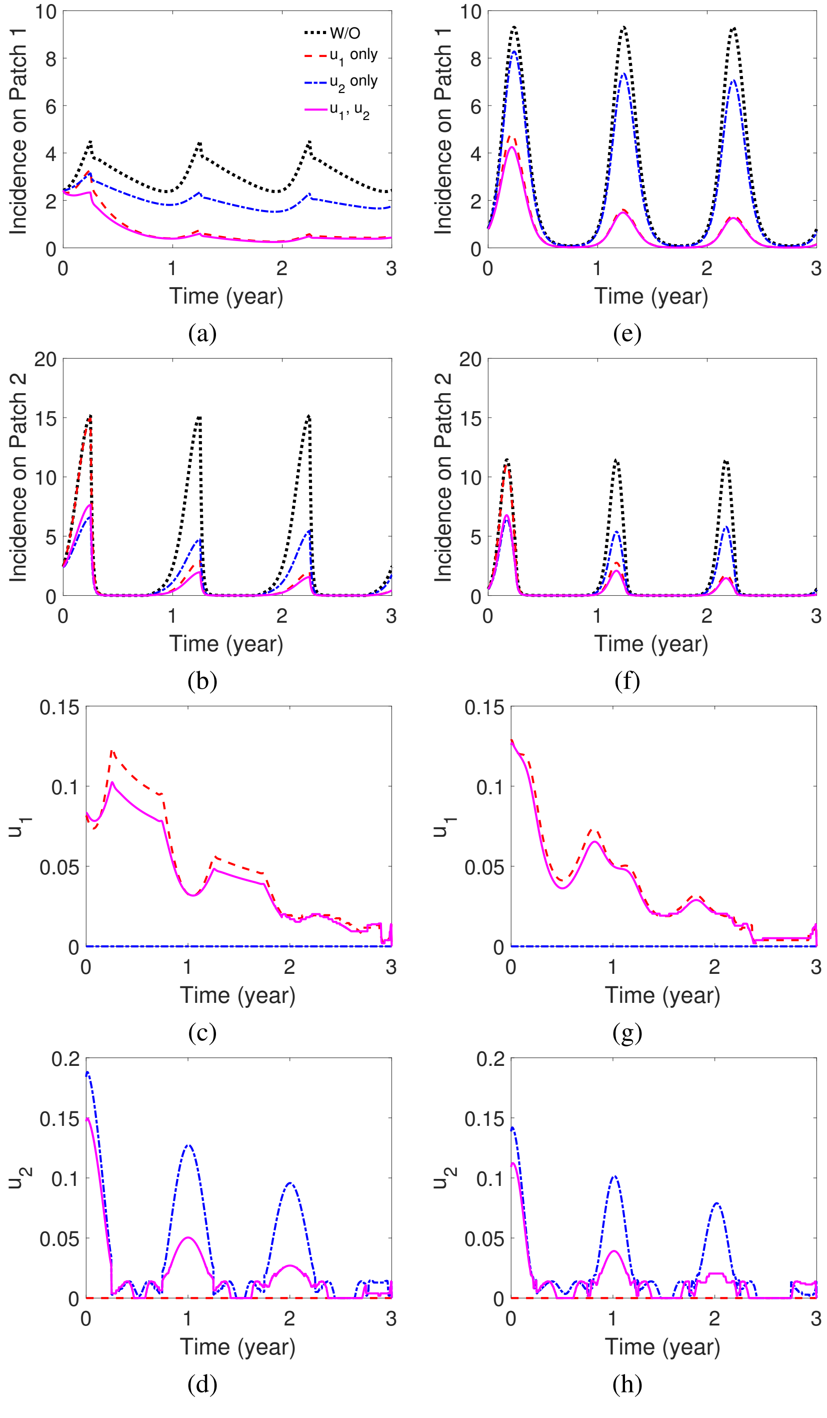

Figure F: The effects of two seasonality scenarios on patch-specific incidence and optimal control functions are compared under weak and asymmetric coupling ($p_{12} = 0.1$, $p_{21} = 0.001$); (a)-(d) the square wave type (S_1) and (e)-(h) the sinusoidal type (S_2).

Optimal control strategies for various weight constants

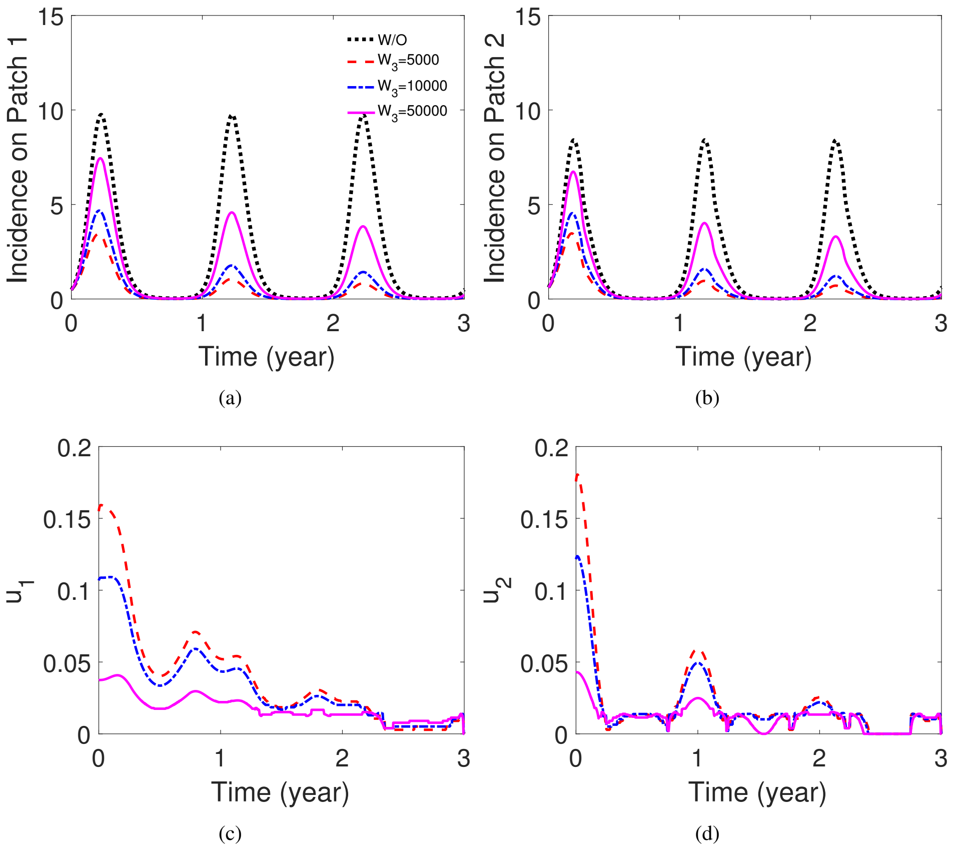

Figure G: The effects of weight constants on patch-specific incidence and optimal control functions are displayed under strong and symmetric coupling ($p_{12} = p_{21} = 0.3$) with the sinusoidal type (S_2) using three different weight constants ($W_3 = W_4 = 5000, 10000, 50000$).

Patch-specific incidence for different patch-sizes

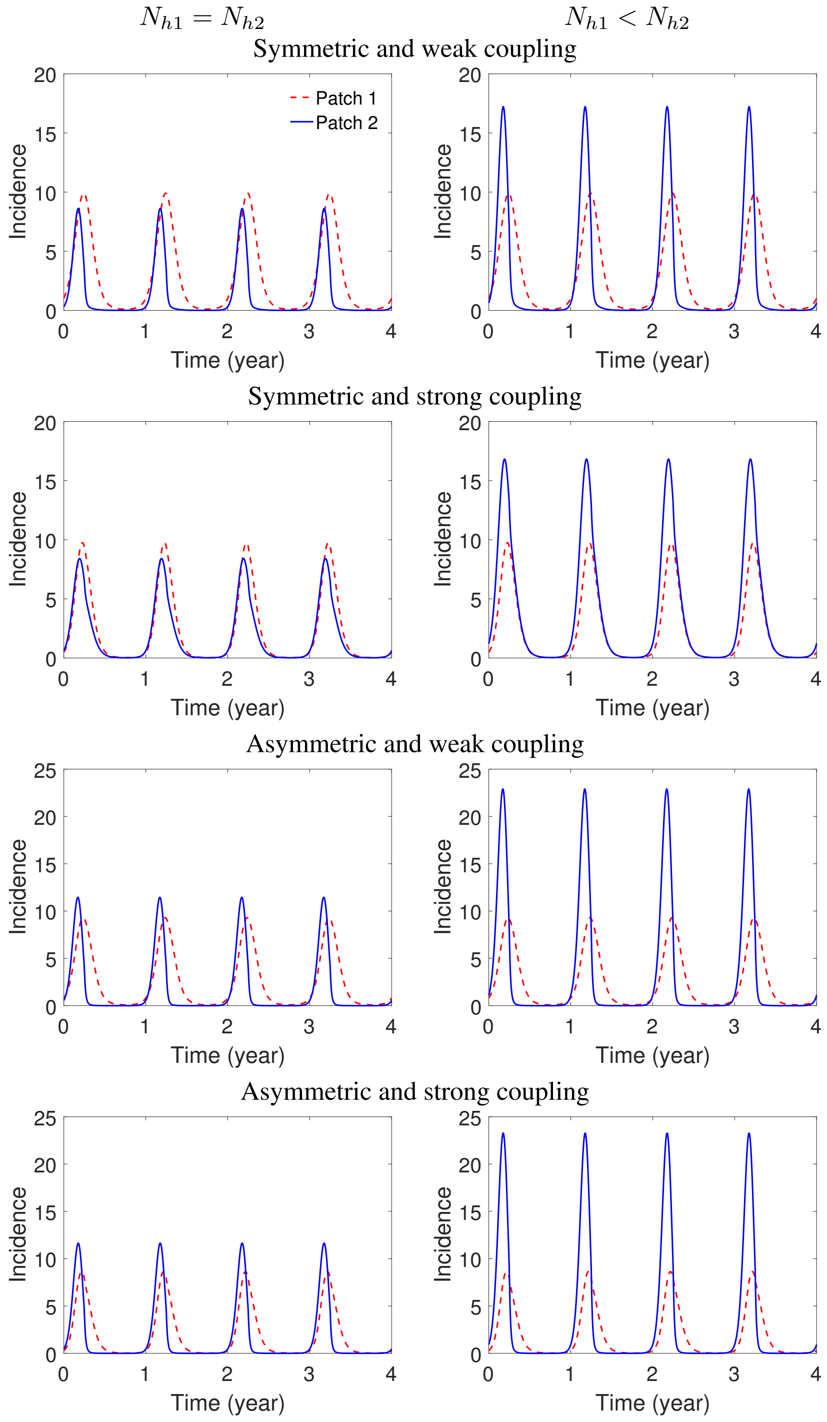

Figure H: The effects of two different patch-sizes on patch-specific incidence are illustrated using the four baseline scenarios: the sinusoidal type (S_2) for symmetric and weak coupling ($p_{12} = p_{21} = 0.01$), symmetric and strong coupling ($p_{12} = 0.3$ and $p_{21} = 0.3$), symmetric and weak coupling ($p_{12} = 0.1$ and $p_{21} = 0.001$) and asymmetric and strong coupling ($p_{12} = 0.3$ and $p_{21} = 0.001$). Patch 1 incidence barely changes, but Patch 2 incidence increases almost twice as N_{h2} is doubled (blue solid in the right panels).

Sensitivity analysis

We perform sensitivity analyses for the parameters that are significantly related to the seasonality of the model. The sensitivity analyses show the relative importance of parameters to dengue transmission dynamics. First, we have chosen patch-specific seasonality parameters, ε_i, μ_v, ξ_i for $i = 1, 2$ (see Table 1). For instance, we vary ε_i (the amplitude of the time-dependent vector birth rate) and μ_v (the background birth/death rates for vectors). Also, the transmission rate functions (both for vectors and hosts) are varied by multiplying a scale factor ξ_i such that $\hat{\beta}_{hi}(t) = \xi_i \beta_{hi}(t)$ and $\hat{\beta}_{vi}(t) = \xi_i \beta_{vi}(t)$. Then, cumulative incidence (CI) for one year is computed when varying one parameter by $\pm 20\%$ from the baseline value while the rests are fixed as the baseline values. Finally, the sensitivity index is defined as $\Delta CI = CI(\mathbf{x}_{new}) - CI(\mathbf{x}_{baseline})$, where $CI(\mathbf{x})$ represents CI using the parameter $\mathbf{x}$. We have normalized the sensitivity index by $\frac{\Delta CI}{CI(\mathbf{x}_{baseline})}$.

Normalized sensitivity index is displayed in Fig 9 using asymmetric and strong coupling ($p_{12} = 0.3$ and $p_{21} = 0.01$) with the sinusoidal seasonality type (S_2). The effect of the amplitude ε_1 (or ε_2) on ΔCI in Patch 1 (Patch 2) is insignificant (small). Clearly, the effects of the transmission rate functions on ΔCI turn out to be the most significant in both patches. The effect of variation of ξ_1 (or ξ_2) is larger in Patch 1 since the transmission rate is higher in Patch 1 (endemic). Note that the vector birth/death rate (μ_v) negatively impact CI, that is, CI decreases when μ_v increases. This is due to the fact that the seasonal reproduction number, $\mathcal{R}_s$ decreases, when μ_v increases (see the formula for $\mathcal{R}_s$ in Section B). Regardless of other coupling scenarios, the effects of parameter changes on CI are qualitatively similar (results are not shown here).

Table 1: Definitions and baseline values of parameters

Parameters	Description	Baseline Value
ε_1	Amplitude of oscillations in vector birth rate in Patch 1	0.1
ε_2	Amplitude of oscillations in vector birth rate in Patch 2	0.2
μ_v	Average vector birth/death rate (days^{-1})	1/14
ξ_i	Scale factor of transmission rates for Patch i	1

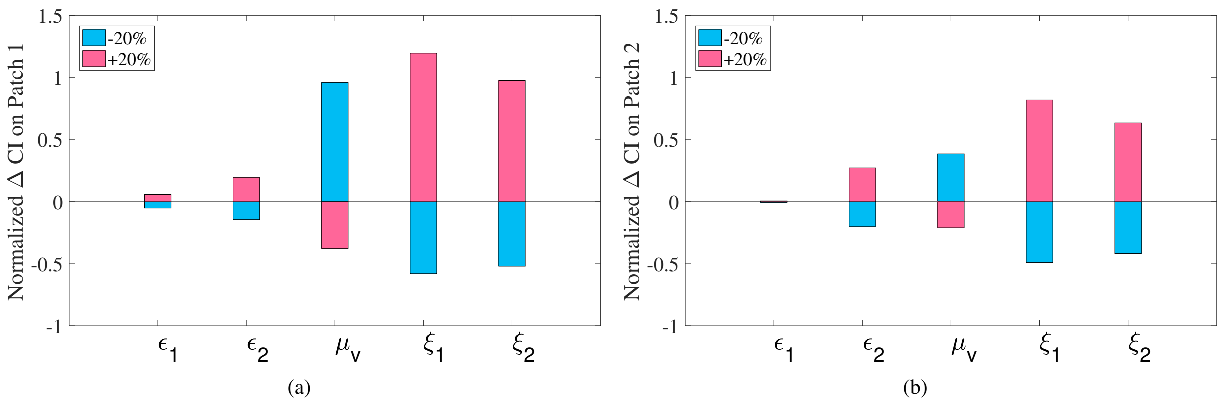

Figure I: Normalized sensitivity index is displayed under asymmetric and strong coupling and the sinusoidal type (S_2) ($p_{12} = 0.3, p_{21} = 0.01$) for (a) Patch 1 (b) Patch 2.

B. The seasonal reproduction number

The seasonal reproduction number is calculated using the methodology (the next generation matrix approach) outlined in [1]. Let $\mathbf{x} = (E_{v1}, I_{v1}, E_{h1}, I_{h1}, E_{v2}, I_{v2}, E_{h2}, I_{h2})^T$. Now, we let $\mathcal{F}_j$ represent the rate of appearance of new infections in the component j's class, thus $\mathcal{F}(\mathbf{x})$ represents all the new infections. The net transition rates out of the corresponding compartment are represented by $\mathcal{V}(\mathbf{x})$.

$$\mathcal{F}(\mathbf{x}) = \begin{pmatrix} \beta_{v1}(p_{11}I_{h1}/N_{h1} + p_{21}I_{h2}/N_{h2})S_{v1} \\ 0 \\ S_{h1}(\beta_{h1}p_{11}I_{v1}/N_{v1} + \beta_{h2}p_{12}I_{v2}/N_{v2}) \\ 0 \\ \beta_{v2}(p_{12}I_{h1}//N_{h1} + p_{22}I_{h2}/N_{h2})S_{v2} \\ 0 \\ S_{h2}(\beta_{h1}p_{21}I_{v1}/N_{v1} + \beta_{h2}p_{22}I_{v2}/N_{v2}) \\ 0 \end{pmatrix}, \mathcal{V}(\mathbf{x}) = \begin{pmatrix} (\kappa+\mu_v)E_{v1} \\ \mu_v I_{v1} - \kappa E_{v1} \\ (\gamma+\mu_h)E_{h1} \\ (\delta+\mu_h)I_{h1} - \gamma E_{h1} \\ (\kappa+\mu_v)E_{v2} \\ \mu_v I_{v2} - \kappa E_{v2} \\ (\gamma+\mu_h)E_{h2} \\ (\delta+\mu_h)I_{h2} - \gamma E_{h2} \end{pmatrix}$$

where we define 8×8 matrices $F = [\frac{\partial \mathcal{F}}{\partial x_j}(\mathbf{x}^*)]$ and $V = [\frac{\partial \mathcal{V}}{\partial x_j}(\mathbf{x}^*)]$ evaluated at the disease-free equilibrium, $\mathbf{x}^*$, which is composed of S_{vi}, S_{hi} for i=1, 2 with the rest being zero. The spectral radius ρ of matrix FV^{-1} yields the basic reproduction number, where

$$F = \begin{bmatrix} 0 & 0 & 0 & \beta_{v1}p_{11}S_{v1}/N_{h1} & 0 & 0 & 0 & \beta_{v1}p_{21}S_{v1}/N_{h2} \\ 0 & 0 & 0 & 0 & 0 & 0 & 0 & 0 \\ 0 & \beta_{h1}p_{11}S_{h1}/N_{v1} & 0 & 0 & 0 & \beta_{h2}p_{12}S_{h1}/N_{v2} & 0 & 0 \\ 0 & 0 & 0 & 0 & 0 & 0 & 0 & 0 \\ 0 & 0 & 0 & \beta_{v2}p_{12}S_{v2}/N_{h1} & 0 & 0 & 0 & \beta_{v2}p_{22}S_{v2}/N_{h2} \\ 0 & 0 & 0 & 0 & 0 & 0 & 0 & 0 \\ 0 & \beta_{h1}p_{21}S_{h2}/N_{v1} & 0 & 0 & 0 & \beta_{h2}p_{22}S_{h2}/N_{v2} & 0 & 0 \\ 0 & 0 & 0 & 0 & 0 & 0 & 0 & 0 \end{bmatrix},$$

and

$$V = \begin{bmatrix} (\kappa+\mu_v) & 0 & 0 & 0 & 0 & 0 & 0 & 0 \\ -\kappa & \mu_v & 0 & 0 & 0 & 0 & 0 & 0 \\ 0 & 0 & (\gamma+\mu_h) & 0 & 0 & 0 & 0 & 0 \\ 0 & 0 & -\gamma & (\delta+\mu_h) & 0 & 0 & 0 & 0 \\ 0 & 0 & 0 & 0 & (\kappa+\mu_v) & 0 & 0 & 0 \\ 0 & 0 & 0 & 0 & -\kappa & \mu_v & 0 & 0 \\ 0 & 0 & 0 & 0 & 0 & 0 & (\gamma+\mu_h) & 0 \\ 0 & 0 & 0 & 0 & 0 & 0 & -\gamma & (\delta+\mu_h) \end{bmatrix},$$

$$V^{-1} = \begin{bmatrix} (\kappa+\mu_v)^{-1} & 0 & 0 & 0 & 0 & 0 & 0 & 0 \\ \frac{\kappa}{(\kappa+\mu_v)\mu_v} & {\mu_v}^{-1} & 0 & 0 & 0 & 0 & 0 & 0 \\ 0 & 0 & (\gamma+\mu_h)^{-1} & 0 & 0 & 0 & 0 & 0 \\ 0 & 0 & \frac{\gamma}{(\gamma+\mu_h)(\delta+\mu_h)} & (\delta+\mu_h)^{-1} & 0 & 0 & 0 & 0 \\ 0 & 0 & 0 & 0 & (\kappa+\mu_v)^{-1} & 0 & 0 & 0 \\ 0 & 0 & 0 & 0 & \frac{\kappa}{(\kappa+\mu_v)\mu_v} & {\mu_v}^{-1} & 0 & 0 \\ 0 & 0 & 0 & 0 & 0 & 0 & (\gamma+\mu_h)^{-1} & 0 \\ 0 & 0 & 0 & 0 & 0 & 0 & \frac{\gamma}{(\gamma+\mu_h)(\delta+\mu_h)} & (\delta+\mu_h)^{-1} \end{bmatrix},$$

and FV^{-1}=

$$\begin{bmatrix} 0 & 0 & \frac{\gamma\beta_{v1}p_{11}S_{v1}}{(\gamma+\mu_h)(\delta+\mu_h)N_{h1}} & \frac{\beta_{v1}p_{11}S_{v1}}{(\delta+\mu_h)N_{h1}} & 0 & 0 & \frac{\gamma\beta_{v1}p_{21}S_{v1}}{(\gamma+\mu_{h2})(\delta+\mu_h)N_{h2}} & \frac{\beta_{v1}p_{21}S_{v1}}{(\delta+\mu_h)N_{h2}} \\ 0 & 0 & 0 & 0 & 0 & 0 & 0 & 0 \\ \frac{\kappa\beta_{h1}p_{11}S_{h1}}{\mu_v(\kappa+\mu_v)N_{v1}} & \frac{\beta_{h1}p_{11}S_{h1}}{\mu_{v1}N_{v1}} & 0 & 0 & \frac{\kappa\beta_{h2}p_{12}S_{h1}}{\mu_v(\kappa+\mu_v)N_{v2}} & \frac{\beta_{h2}p_{12}S_{h1}}{\mu_v N_{v2}} & 0 & 0 \\ 0 & 0 & 0 & 0 & 0 & 0 & 0 & 0 \\ 0 & 0 & \frac{\gamma\beta_{v2}p_{12}S_{v2}}{(\gamma+\mu_h)(\delta+\mu_h)N_{h1}} & \frac{\beta_{v2}p_{12}S_{v2}}{(\delta+\mu_h)N_{h1}} & 0 & 0 & \frac{\gamma\beta_{v2}p_{22}S_{v2}}{(\gamma+\mu_h)(\delta+\mu_h)N_{h2}} & \frac{\beta_{v2}p_{22}S_{v2}}{(\delta+\mu_h)N_{h2}} \\ 0 & 0 & 0 & 0 & 0 & 0 & 0 & 0 \\ \frac{\kappa\beta_{h1}p_{21}S_{h2}}{\mu_v(\kappa+\mu_v)N_{v1}} & \frac{\beta_{h1}p_{21}S_{h2}}{\mu_v N_{v1}} & 0 & 0 & \frac{\kappa\beta_{h2}p_{22}S_{h2}}{\mu_v(\kappa+\mu_v)N_{v2}} & \frac{\beta_{h2}p_{22}S_{h2}}{\mu_v N_{v2}} & 0 & 0 \\ 0 & 0 & 0 & 0 & 0 & 0 & 0 & 0 \end{bmatrix}.$$

Thus, the globally seasonal reproduction number for the two-patch system is

$$\mathcal{R}_s = \sqrt{\frac{\kappa\gamma}{2C}(\phi_1 + \sqrt{\phi_2})},$$

where

$$\phi_1 = B_1 + B_2$$
$$\phi_2 = B_3(A_1 + A_2 + A_3)$$

$$\begin{aligned}
C &= \mu_v\left(\kappa+\mu_v\right)\left(\gamma+\mu_h\right)\left(\delta+\mu_h\right)\\
B_1 &= (\beta_{h1}\beta_{v1}p_{11}^2 S_{h1}S_{v1})/(N_{h1}N_{v1}) + (\beta_{h1}\beta_{v1}p_{21}^2 S_{h2}S_{v1})/(N_{h2}N_{v1})\\
B_2 &= (\beta_{h2}\beta_{v2}p_{12}^2 S_{h1}S_{v2})/(N_{h1}N_{v2}) + (\beta_{h2}\beta_{v2}p_{22}^2 S_{h2}S_{v2})/(N_{h2}N_{v2})\\
B_3 &= 1/(N_{h1}^2N_{h2}^2N_{v1}^2N_{v2}^2)\\
A_1 &= \beta_{h1}^2\beta_{v1}^2N_{v2}^2(N_{h2}p_{11}^2S_{h1} + N_{h1}p_{21}^2S_{h2})^2S_{v1}^2\\
A_2 &= 2\beta_{h1}\beta_{h2}\beta_{v1}\beta_{v2}N_{v1}N_{v2}(N_{h2}^2p_{11}^2p_{12}^2S_{h1}^2 + N_{h1}N_{h2}(4p_{11}p_{12}p_{21}p_{22} - p_{12}^2p_{21}^2 - p_{11}^2p_{22}^2)S_{h1}S_{h2}\\
&+ N_{h1}^2p_{21}^2p_{22}^2S_{h2}^2)S_{v1}S_{v2}\\
A_3 &= \beta_{h2}^2\beta_{v2}^2N_{v1}^2(N_{h2}p_{12}^2S_{h1} + N_{h1}p_{22}^2S_{h2})^2S_{v2}^2.
\end{aligned}$$

C. Optimal control framework

Next, the optimal control problem for the two-patch model is formulated to minimize the proportions of infected vectors and humans in both patches for a finite time interval at a minimal cost of implementation. We define our objective functional as follows

$$J(u_1(t),u_2(t)) = \int_0^{t_f} W_1(I_{h1}(t)+I_{v1}(t)) + W_2(I_{h2}(t)+I_{v2}(t)) + \frac{1}{2}W_3u_1^2(t) + \frac{1}{2}W_4u_2^2(t)dt.$$

Then, we seek an optimal pair (U^*, X^*) such that

$$J(U^*) = min\{J(U)|U \in \Omega\},$$

where $\Omega = \{(u_i(t) \in (L^1(0,t_f))^2 \parallel a \le u_i(t) \le b, t \in [0,t_f], i = 1,2\}$ subject to the state equations with $X = (S_{v1}, E_{v1}, I_{v1}, S_{h1}, E_{h1}, I_{h1}, R_{h1}, S_{v2}, E_{v2}, I_{v2}, S_{h2}, E_{h2}, I_{h2}, R_{h2})$ and $U = (u_1, u_2)$. The existence of optimal controls is guaranteed by standard results of optimal control theory [2]. The necessary conditions of optimal solutions are derived from Pontryagin's Maximum Principle [3]. This principle converts the system into the problem of minimizing the Hamiltonian H given by

$$\begin{aligned}
H = \quad & W_1(I_{h1}(t)+I_{v1}(t)) + W_2(I_{h2}(t)+I_{v2}(t)) + \frac{1}{2}W_3u_1^2(t) + \frac{1}{2}W_4u_2^2(t)\\
&+\lambda_1[\mu_{v1}(t)N_{v1} - \beta_{v1}(t)(1-u_1(t))(p_{11}I_{h1}/N_{h1} + p_{21}I_{h2}/N_{h2})S_{v1} - \mu_vS_{v1}]\\
&+\lambda_2[\beta_{v1}(t)(1-u_1(t))(p_{11}I_{h1}/N_{h1} + p_{21}I_{h2}/N_{h2})S_{v1} - \mu_vE_{v1} - \kappa E_{v1}]\\
&+\lambda_3[\kappa E_{v1} - \mu_vI_{v1}]\\
&+\lambda_4[\mu_hN_{h1} - S_{h1}(\beta_{h1}(t)(1-u_1(t))p_{11}I_{v1}/N_{v1} + \beta_{h2}(t)(1-u_2(t))p_{12}I_{v2}/N_{v2}) - \mu_hS_{h1}]\\
&+\lambda_5[S_{h1}(\beta_{h1}(t)(1-u_1(t))p_{11}I_{v1}/N_{v1} + \beta_{h2}(t)(1-u_2(t))p_{12}I_{v2}/N_{v2}) - \gamma E_{h1} - \mu_hE_{h1}]\\
&+\lambda_6[\gamma E_{h1} - \delta I_{h1} - \mu_hI_{h1}]\\
&+\lambda_7[\mu_{v2}(t)N_{v2} - \beta_{v2}(t)(1-u_2(t))(p_{12}I_{h1}/N_{h1} + p_{22}I_{h2}/N_{h2})S_{v2} - \mu_vS_{v2}]\\
&+\lambda_8[\beta_{v2}(t)(1-u_2(t))(p_{12}I_{h1}//N_{h1} + p_{22}I_{h2}/N_{h2})S_{v2} - \mu_vE_{v2} - \kappa E_{v2}]\\
&+\lambda_9[\kappa E_{v2} - \mu_vI_{v2}]\\
&+\lambda_{10}[\mu_hN_{h2} - S_{h2}(\beta_{h1}(t)(1-u_1(t))p_{21}I_{v1}/N_{v1} + \beta_{h2}(t)(1-u_2(t))p_{22}I_{v2}/N_{v2}) - \mu_hS_{h2}]\\
&+\lambda_{11}[S_{h2}(\beta_{h1}(t)(1-u_1(t))p_{21}I_{v1}/N_{v1} + \beta_{h2}(t)(1-u_2(t))p_{22}I_{v2}/N_{v2}) - \gamma E_{h2} - \mu_hE_{h2}]\\
&+\lambda_{12}[\gamma E_{h2} - \delta I_{h2} - \mu_hI_{h2}]. \qquad (1)
\end{aligned}$$

From this Hamiltonian and Pontryagin's Maximum Principle [3], we obtain the following theorem:

Theorem 1 *There exist optimal controls $U^*(t)$ and corresponding state solutions $X^*(t)$ that minimize $J(U)$ over Ω. In order for the above statement to be true, it is necessary that there exist continuous functions $\lambda_i(t)$ such that*

$$
\begin{aligned}
\lambda_1^{'} &= \lambda_1[-\mu_{v1}(t)+\beta_{v1}(t)(1-u_1(t))(p_{11}I_{h1}/N_{h1}+p_{21}I_{h2}/N_{h2})+\mu_v] \\
&\quad -\lambda_2\beta_{v1}(t)(1-u_1(t))(p_{11}I_{h1}/N_{h1}+p_{21}I_{h2}/N_{h2}) \\
&\quad +(\lambda_5-\lambda_4)S_{h1}\big(\beta_{h1}(t)(1-u_1(t))p_{11}I_{v1}/N_{v1}^2\big) \\
&\quad +(\lambda_{11}-\lambda_{10})S_{h2}\big(\beta_{h1}(t)(1-u_1(t))p_{21}I_{v1}/N_{v1}^2\big) \\
\lambda_2^{'} &= -\lambda_1\mu_{v1}+\lambda_2(\mu_v+\kappa)-\lambda_3\kappa \\
&\quad +(\lambda_5-\lambda_4)S_{h1}\big(\beta_{h1}(t)(1-u_1(t))p_{11}I_{v1}/N_{v1}^2\big) \\
&\quad +(\lambda_{11}-\lambda_{10})S_{h2}\big(\beta_{h1}(t)(1-u_1(t))p_{21}I_{v1}/N_{v1}^2\big) \\
\lambda_3^{'} &= -W_1-\lambda_1\mu_{v1}+\lambda_3\mu_v+(\lambda_4-\lambda_5)S_{h1}\beta_{h1}(t)(1-u_1(t))p_{11}(S_{v1}+E_{v1})/N_{v1}^2 \\
&\quad +(\lambda_{10}-\lambda_{11})S_{h2}\beta_{h1}(t)(1-u_1(t))p_{21}(S_{v1}+E_{v1})/N_{v1}^2 \\
\lambda_4^{'} &= (\lambda_4-\lambda_5)\big(\beta_{h1}(t)(1-u_1(t))p_{11}I_{v1}/N_{v1}+\beta_{h2}(t)(1-u_2(t))p_{12}I_{v2}/N_{v2}\big)+\lambda_4\mu_h \\
\lambda_5^{'} &= \lambda_5(\gamma+\mu_h)-\lambda_6\gamma \\
\lambda_6^{'} &= -W_1+(\lambda_1-\lambda_2)\beta_{v1}(t)(1-u_1(t))(p_{11}/N_{h1})S_{v1}+\lambda_6(\delta+\mu_h) \\
&\quad +(\lambda_7-\lambda_8)\beta_{v2}(t)(1-u_2(t))(p_{12}/N_{h1})S_{v2} \\
\lambda_7^{'} &= (\lambda_5-\lambda_4)S_{h1}\big(\beta_{h2}(t)(1-u_2(t))p_{12}I_{v2}/N_{v2}^2\big) \\
&\quad +(\lambda_{11}-\lambda_{10})S_{h2}\big(\beta_{h2}(t)(1-u_2(t))p_{22}I_{v2}/N_{v2}^2\big) \\
&\quad +\lambda_7[-\mu_{v2}+\beta_{v2}(t)(1-u_2(t))(p_{12}I_{h1}/N_{h1}+p_{22}I_{h2}/N_{h2})+\mu_v] \\
&\quad -\lambda_8\beta_{v2}(t)(1-u_2(t))(p_{12}I_{h1}/N_{h1}+p_{22}I_{h2}/N_{h2}) \\
\lambda_8^{'} &= (\lambda_5-\lambda_4)S_{h1}\big(\beta_{h2}(t)(1-u_2(t))p_{12}I_{v2}/N_{v2}^2\big) \\
&\quad +(\lambda_{11}-\lambda_{10})S_{h2}\big(\beta_{h2}(t)(1-u_2(t))p_{22}I_{v2}/N_{v2}^2\big) \\
&\quad -\lambda_7\mu_{v2}+\lambda_8(\mu_v+\kappa)-\lambda_9\kappa \\
\lambda_9^{'} &= -W_2+(\lambda_4-\lambda_5)S_{h1}\beta_{h2}(t)(1-u_2(t))p_{12}(S_{v2}+E_{v2})/N_{v2}^2 \\
&\quad +(\lambda_{10}-\lambda_{11})S_{h2}\beta_{h2}(t)(1-u_2(t))p_{22}(S_{v2}+E_{v2})/N_{v2}^2 \\
&\quad -\lambda_7\mu_{v2}+\lambda_9\mu_v \\
\lambda_{10}^{'} &= (\lambda_{10}-\lambda_{11})\big(\beta_{h1}(t)(1-u_1(t))p_{21}I_{v1}/N_{v1}+\beta_{h2}(t)(1-u_2(t))p_{22}I_{v2}/N_{v2}\big)+\lambda_{10}\mu_h \\
\lambda_{11}^{'} &= \lambda_{11}(\gamma+\mu_h)-\lambda_{12}\gamma \\
\lambda_{12}^{'} &= -W_2+(\lambda_1-\lambda_2)\beta_{v1}(t)(1-u_1(t))(p_{21}/N_{h2})S_{v1}+\lambda_{12}(\delta+\mu_h) \\
&\quad +(\lambda_7-\lambda_8)\beta_{v2}(t)(1-u_2(t))(p_{22}/N_{h2})S_{v2}
\end{aligned}
$$

with the transversality conditions $\lambda_i(t_f)=0$ *for* $i=1,...,12$ *and the optimality conditions*

$$
\begin{aligned}
u_1^* &= min\{max\{a,\beta_{v1}S_{v1}\sum_{j=1}^{2}p_{j1}\frac{I_{hj}}{N_{hj}}\frac{\lambda_2-\lambda_1}{W_3}+\beta_{h1}p_{11}S_{h1}\frac{I_{v1}}{N_{v1}}\frac{\lambda_5-\lambda_4}{W_3}+\beta_{h1}p_{21}S_{h2}\frac{I_{v1}}{N_{v1}}\frac{\lambda_{11}-\lambda_{10}}{W_3}\},b\} \qquad (2) \\
u_2^* &= min\{max\{a,\beta_{v2}\beta_{v2}S_{v2}\sum_{j=1}^{2}p_{j2}\frac{I_{hj}}{N_{hj}}\frac{\lambda_8-\lambda_7}{W_4}+\beta_{h2}p_{12}S_{h1}\frac{I_{v2}}{N_{v2}}\frac{\lambda_5-\lambda_4}{W_4}+\beta_{h2}p_{22}S_{h2}\frac{I_{v2}}{N_{v2}}\frac{\lambda_{11}-\lambda_{10}}{W_4}\},b\}.
\end{aligned}
$$

Proof The existence of optimal controls follows from Corollary 4.1 of [2] since the integrand of J is a convex function of $U(t)$ and the the state system satisfies the *Lipschitz* property with respect to the state variables. The following can be derived from the Pontryagin's Maximum Principle [3]:

$$
\begin{aligned}
\frac{d\lambda_1(t)}{dt} &= -\frac{\partial H}{\partial S_{v1}}, \; \frac{d\lambda_2(t)}{dt} = -\frac{\partial H}{\partial E_{v1}}, \; \frac{d\lambda_3(t)}{dt} = -\frac{\partial H}{\partial I_{v1}}, \\
\frac{d\lambda_4(t)}{dt} &= -\frac{\partial H}{\partial S_{h1}}, \; \frac{d\lambda_5(t)}{dt} = -\frac{\partial H}{\partial E_{h1}}, \; \frac{d\lambda_6(t)}{dt} = -\frac{\partial H}{\partial I_{h1}}, \\
\frac{d\lambda_7(t)}{dt} &= -\frac{\partial H}{\partial S_{v2}}, \; \frac{d\lambda_8(t)}{dt} = -\frac{\partial H}{\partial E_{v2}}, \; \frac{d\lambda_9(t)}{dt} = -\frac{\partial H}{\partial I_{v2}}, \\
\frac{d\lambda_{10}(t)}{dt} &= -\frac{\partial H}{\partial S_{h2}}, \; \frac{d\lambda_{11}(t)}{dt} = -\frac{\partial H}{\partial E_{h2}}, \; \frac{d\lambda_{12}(t)}{dt} = -\frac{\partial H}{\partial I_{h2}},
\end{aligned}
$$

with $\lambda_i(t_f) = 0$ for $i = 1, ..., 12$ and evaluated at the optimal controls and corresponding states, which results in the adjoint system. The Hamiltonian H is minimized with respect to the controls, so we differentiate H with respect to u_i on the set Ω, respectively, giving the following optimality conditions:

$$
\begin{aligned}
0 = \frac{\partial H}{\partial u_1} &= W_3 u_1 + (\lambda_1 - \lambda_2)\beta_{v1} S_{v1} \sum_{j=1}^{2} p_{j1} I_{hj}/N_{hj} + (\lambda_4 - \lambda_5)\beta_{h1} p_{11} S_{h1} I_{v1}/N_{v1} \\
&+ (\lambda_{10} - \lambda_{11})\beta_{h1} p_{21} S_{h2} I_{v1}/N_{v1} \\
0 = \frac{\partial H}{\partial u_2} &= W_4 u_2 + (\lambda_4 - \lambda_5)\beta_{h2} p_{12} S_{h1} I_{v2}/N_{v2} + (\lambda_7 - \lambda_8)\beta_{v2} S_{v2} \sum_{j=1}^{2} p_{j2} I_{hj}/N_{hj} \\
&+ (\lambda_{10} - \lambda_{11})\beta_{h2} p_{22} S_{h2} I_{v2}/N_{v2}. \qquad (3)
\end{aligned}
$$

Solving for $u_i^*(t)$ we obtain

$$
\begin{aligned}
u_1^* &= \beta_{v1} S_{v1} \sum_{j=1}^{2} p_{j1} \frac{I_{hj}}{N_{hj}} \frac{\lambda_2 - \lambda_1}{W_3} + \beta_{h1} p_{11} S_{h1} \frac{I_{v1}}{N_{v1}} \frac{\lambda_5 - \lambda_4}{W_3} + \beta_{h1} p_{21} S_{h2} \frac{I_{v1}}{N_{v1}} \frac{\lambda_{11} - \lambda_{10}}{W_3}, \qquad (4) \\
u_2^* &= \beta_{v2} \beta_{v2} S_{v2} \sum_{j=1}^{2} p_{j2} \frac{I_{hj}}{N_{hj}} \frac{\lambda_8 - \lambda_7}{W_4} + \beta_{h2} p_{12} S_{h1} \frac{I_{v2}}{N_{v2}} \frac{\lambda_5 - \lambda_4}{W_4} + \beta_{h2} p_{22} S_{h2} \frac{I_{v2}}{N_{v2}} \frac{\lambda_{11} - \lambda_{10}}{W_4}.
\end{aligned}
$$

We obtain the optimality conditions by using the standard argument for bounds $a \leq u_i(t) \leq b$ for $i = 1, 2$.

References

[1] Van den Driessche P, Watmough J. Reproduction numbers and sub-threshold endemic equilibria for compartmental models of disease transmission. Mathematical biosciences. 2002;180(1):29–48.

[2] Fleming WH, Rishel RW. Deterministic and stochastic optimal control. vol. 1. Springer Science & Business Media; 2012.

[3] Pontryagin LS, Boltyanskii VG, Gamkrelidze RV, Mishchenkoi EF. The Mathematical Theory of Optimal Processes. Wiley, New Jersey; 1962.
